# Multi-scale ensemble properties of the *Escherichia coli* RNA degradosome

**DOI:** 10.1111/mmi.14800

**Published:** 2021-09-25

**Authors:** Tom Dendooven, Giulia Paris, Alexander V. Shkumatov, Md. Saiful Islam, Alister Burt, Marta A. Kubańska, Tai Yuchen Yang, Steven W. Hardwick, Ben F. Luisi

**Affiliations:** 1 Department of Biochemistry, University of Cambridge, Cambridge, UK; 2 Center for Structural Biology, Vlaams Instituut voor Biotechnologie, Brussels, Belgium; 3 Structural Biology Brussels, Department of Bioengineering Sciences, Vrije Universiteit Brussel, Brussels, Belgium; 4 Institut de Biologie Structurale, Université Grenoble Alpes, CEA, CNRS, IBS, Grenoble, France

**Keywords:** colloidal polymer, intrinsically disordered protein, post-transcriptional gene regulation, ribonuclease, riboregulation, RNA condensates, RNA metabolism, RNA surveillance, RNase E

## Abstract

In organisms from all domains of life, multi-enzyme assemblies play central roles in defining transcript lifetimes and facilitating RNA-mediated regulation of gene expression. An assembly dedicated to such roles, known as the RNA degradosome, is found amongst bacteria from highly diverse lineages. About a fifth of the assembly mass of the degradosome of *Escherichia coli* and related species is predicted to be intrinsically disordered – a property that has been sustained for over a billion years of bacterial molecular history and stands in marked contrast to the high degree of sequence variation of that same region. Here, we characterize the conformational dynamics of the degradosome using a hybrid structural biology approach that combines solution scattering with ad hoc ensemble modelling, cryo-electron microscopy, and other biophysical methods. The *E. coli* degradosome can form punctate bodies in vivo that may facilitate its functional activities, and based on our results, we propose an electrostatic switch model to account for the propensity of the degradosome to undergo programmable puncta formation.

## 1 Introduction

The fate of cellular mRNAs often has as much importance in the control of gene expression as the processes of transcription and translation themselves (Felden & Paillard, 2017). The coordination of RNA lifetime, together with adjustments to synthesis rates, maintains transcript homeostasis, enables rapid change of gene expression, and signals cellular status (Adler & Alon, 2018; Bresson & Tollervey, 2018; Sun et al., 2012; Haimovich et al., 2013; Pérez-Ortín et al., 2007; Schmid & Jensen, 2018; Tudek et al., 2018). RNA degradation and processing in prokaryotes has been extensively studied using model organisms such as the Gram-negative *Escherichia coli* and the Gram-positive *Bacillus subtilis* that represent highly divergent bacterial lineages. RNA decay in these bacteria is centered around multi-enzyme complexes, known as RNA degradosomes, that have arisen through both convergent and divergent evolution, indicating that they have met a common biological requirement to control mRNA lifetime. While the composition and organization of bacterial RNA degradosomes vary markedly, their widespread occurrence amongst diverse bacteria emphasizes their functional importance, as does also their functional parallels with the eukaryotic exosome and RISC (RNA-Induced Silencing Complex) machinery (Bandyra & Luisi, 2018; Dendooven et al., 2020).

**Figure 1:**
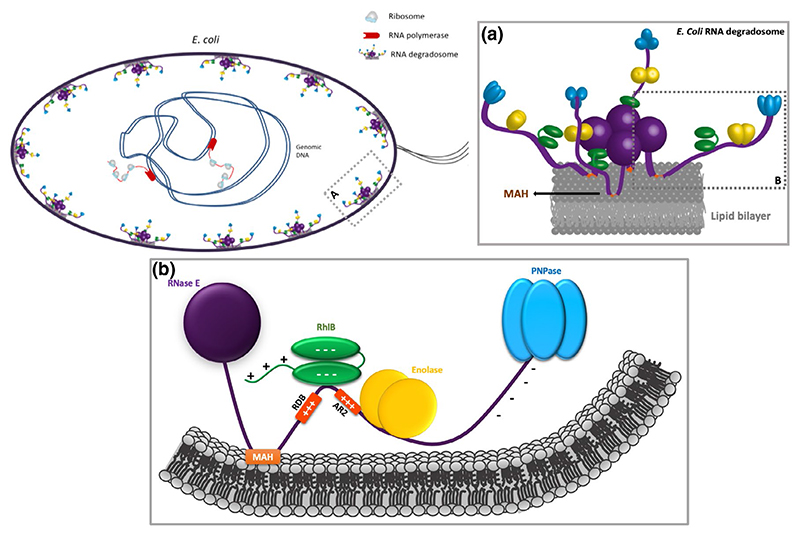
Schematic of the membrane association and architecture of *Escherichia coli* RNA degradosome. (a) The degradosome is a homo-tetrameric multi-enzyme assembly that is tethered to the bacterial inner membrane via an amphipathic Membrane Attachment Helix (MAH) found in the C-terminal half of RNase E. The scaffold of the degradosome is the endoribonuclease RNase E, which has an N-terminal catalytic domain, responsible for initial RNA substrate cleavage, and a C-terminal scaffold domain predicted to be intrinsically disordered. Adapted from Dendooven and Luisi (2017) and Bandyra et al. (2013). (b) Expanded view of the region indicated with the rectangle in panel (a). The scaffold domain is a mosaic of microdomains involved in recruiting other degradosome components and RNA substrates. Two RNA binding sites (RBD and AR2) capture RNA substrates, and there are interaction sites for the DEAD-box helicase RhlB, the glycolytic enzyme enolase and the exoribonuclease PNPase (Bandyra et al., 2013; Dendooven & Lavigne, 2019; Dendooven & Luisi, 2017). The electropositive regions are color-coded according to Table 1. While the electropositive regions of the scaffold domain are expected to interact with the negatively charged groups of the membrane, the PNPase interaction segment (850-1061) is electronegative. Therefore, it is likely that the latter region of the C-terminal domain of RNase E would be repelled away from the membrane and may impede self-interactions. Binding nucleic acid would change the charge distribution and might enable shared interactions with the RNA between adjacent degradosome assemblies

The *E. coli* RNA degradosome is a complex molecular machine and main actor in steady state turnover of the cellular mRNA pool and RNA-mediated post-transcriptional regulation of gene expression. Discovered by Carpousis et al. (1994), the degradosome has the conserved exoribonuclease RNase E at its core (Figure 1). The enzyme has a structured N-terminal domain, corresponding to half of the protein mass, which oligomerizes into functional tetramers and provides the active site that cleaves RNA substrates hydrolytically (Callaghan et al., 2005). The C-terminal half of RNase E serves as a scaffold for recruitment of other enzymes of the degradosome (Bandyra et al., 2013; Bruce et al., 2018). The canonical components of the *E. coli* RNA degradosome are the phosphorolytic exoribonuclease PNPase (polynucleotide phosphorylase), the glycolytic enzyme enolase, and the DEAD-box helicase RhlB that unfolds secondary structures in RNA substrates, or remodels protein-RNA complexes, to make cleavage sites accessible (Py et al., 1996). Although the detailed role of enolase in the *E*. *coli* degradosome assembly is still elusive, its interaction with RNase E is required for cell division control under anaerobic conditions (Morita et al., 2004; Murashko & Lin-Chao, 2017). More generally, it has become clear that a canonical RNA degradosome, both in Gram-negative and Gram-positive bacteria, consists of at least a ribonuclease and a facilitating helicase of the ATP-dependent DEAD-box family (Aït-Bara & Carpousis, 2015). Often bacterial degradosomes have more than one type of ribonuclease, each with different enzymatic activities, and include a metabolic enzyme (Lehnik-Habrink et al., 2011; Roux et al., 2011; Voss et al., 2014).

The scaffold domain of the *E. coli* RNA degradosome contains a short amphipathic α-helical domain that interacts with the *E. coli* inner membrane (Figure 1a,b). The resulting membrane localization of the degradosome adds a spatial layer to post-transcriptional gene regulation (Mackie, 2013). Disrupting the RNase E membrane attachment helix in *E. coli* generates a cytoplasmic form of the degradosome, and is associated with severely disrupted cellular mRNA turnover, slowing it down globally, yet increasing turnover of exposed mRNAs that are not engaged by ribosomes (Hadjeras et al., 2019). The RNase E scaffold domain also contains two RNA binding sites (AR2 and RBD) that capture substrates and cooperate with RhlB to assist in substrate unwinding (Chandran et al., 2007; Garrey et al., 2009; Khemici & Carpousis, 2004; Leroy et al., 2002). These RNA-binding segments are enriched in basic residues, in striking contrast to the acidic nature of the other parts of RNase E (Figure 1b and Table 1). The two RNA binding sites, together with RhlB, have been shown to interact with translating ribosomes (Tsai et al., 2012). Interaction of the RNA degradosome with ribosomes might trigger mRNA degradation as a scavenging process (Bayas et al., 2018; Deana & Belasco, 2005; Dreyfus, 2009; Hamouche et al., 2021; Iost & Dreyfus, 1995), with certain analogy to RNA surveillance process in eukaryotes mediated by the Ski-helicase complex (Schmidt et al., 2016). A plausible scenario is that the close proximity of the RNA degradosome to the translational machine prevents the translation of aberrant transcripts and rescues stalled ribosomal assemblies as part of bacterial RNA surveillance.

Most of the C-terminal domain of RNase E is predicted to be intrinsically disordered, and the interactions of the scaffold domain of RNase E with partner proteins, RNA and the cytoplasmic membrane are mediated by microdomains that are conserved segments of 20–70 amino acids with predicted structural propensity (Górna et al., 2012; Figure 1b and Table 1). While the evolving microdomains experience less restrictive pressure to fold into a globally stable and functional structure (Marcaida et al., 2006), it is clear that there are evolutionary drives that have sustained intrinsically disordered regions (IDRs) in the scaffold domain (Aït-Bara & Carpousis, 2015). One such function may be the propensity to drive the degradosome into microscopic condensates in the presence of RNA (Al-Husini et al., 2018, 2020; Strahl et al., 2015). The degradosome shares this property with many other RNA binding proteins from all domains of life, which are also observed to undergo liquid-liquid phase separation (LLPS) in the presence of RNA (Banani et al., 2017; Boeynaems et al., 2018; Cornejo et al., 2014; Lin et al., 2015; Protter et al., 2018). The environment within the separated phase can alter the stability of substrate RNA secondary structures (Nott et al., 2016), and the physicochemical conditions and concentrated enzymatic activities in these bodies may define the fate of substrate RNAs, whether it is turnover or storage (Guzikowski et al., 2019). The RNA substrates captured in the *Caulobacter crescentus* RNA degradosome condensates are predominantly small regulatory RNAs, antisense RNAs and poorly translated mRNAs (Al-Husini et al., 2020), while ribosomal RNAs (rRNAs) and transfer RNAs (tRNAs) are underrepresented. The ribonucleoprotein bodies stimulate RNA decay of target RNAs and complete mRNA turnover, preventing accumulation of potentially harmful endo-cleaved degradation intermediates.

Many studies, both functional and structural, have been carried out on individual components or small sub-assemblies of the degradosome. However, studying the degradosome as a whole is very challenging and requires tailored methods. Here, we describe an integrative approach to studying the degradosome and its subassemblies using X-ray solution scattering, cryo-EM single particle analysis and cryo-electron tomography. These experiments probe the intrinsically disordered character of the degradosome, its membrane association and interactions with ribosomes or ribosomal subunits. We show that the canonical enzymes of the RNA degradosome are arranged like beads on a string, and that the catalytic core itself is conformationally heterogeneous. Upon engaging an RNA substrate, the degradosome forms a more compacted but disordered ensemble. We also visualize how the recognition core of the RNA degradosome can engage the 30S ribosomal subunit in an assembly that may support RNA quality control. Our findings give insight into rules that can switch the degradosome into a condensed phase.

## 2 Results

### 2.1 Conformational flexibility of the catalytic core of RNase E revealed by single particle cryo-EM

**Figure 2:**
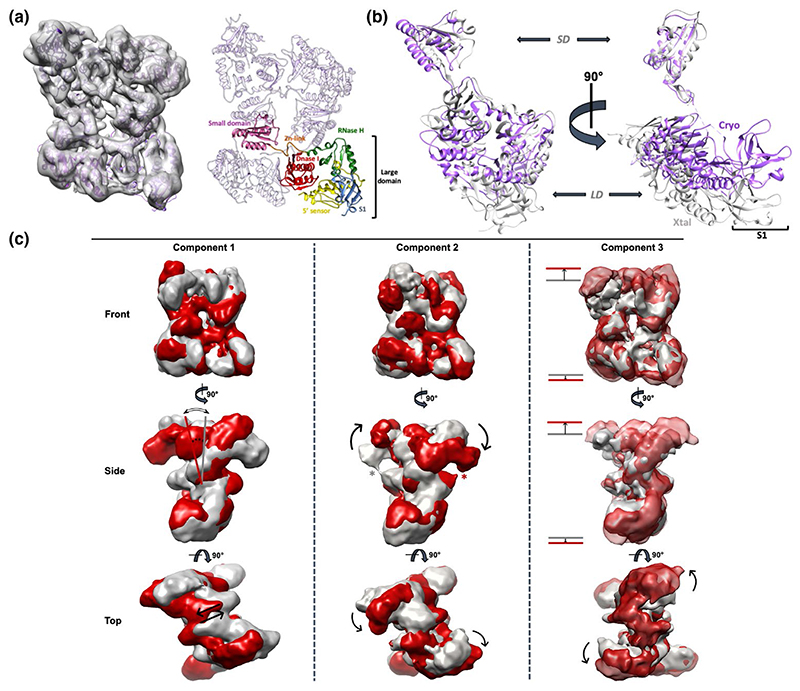
A flexible quaternary structure for the RNase E catalytic core. (a) 3D refinement of 38K particles results in a 7.8 Å reconstruction of the tetrameric RNase E core (left). A model with color-coded domains is presented on the right. (b) Significant conformational reorganizations are necessary for the RNase E (1-529) crystal structure (pdb 2VMK) to fit the cryo-EM map. In particular, there is a significant conformational change between the RNase E large domains, with a substantial reorganization of the S1 domain. Cryo, cryo-EM structure; Xtal, X-ray crystallography structure; *SD*, small domain; *LD*, large domain. (c) Three-dimensional variation analysis reveals three main modes of molecular motion within the RNase E tetramer. Variability components 1, 2 and 3 correspond to a “rocking”, “rolling” and “breathing” motion, respectively, between low-energy states of the RNase E core. * Highlights a potential interaction between the S1 and small domains of adjacent RNase E protomers (Component 2, side view). The red and grey maps are densities of two extreme conformational states of the indicated mode

Crystallographic studies reveal that the catalytic core of RNase E (1-529) can undergo significant conformational change upon RNA binding (Bandyra et al., 2018; Callaghan et al., 2005; Koslover et al., 2008). The isolated catalytic core (without RNA) was studied by cryo-EM to characterize its endogenous conformational heterogeneity. The catalytic core aggregated and dissociated using standard grid preparation protocols and grid support layers. To prevent this, the zwitterionic detergent CHAPSO was added prior to grid freezing, which significantly improved the quality of the specimen and resulted in a homogeneous set of particles (Supplementary Figure S1a). 2D classification of ~180,000 particles produced well resolved 2D class averages with apparent C2 symmetry (Supplementary Figure S1b), and after several rounds of 3D classification ~38,000 particles were used for 3D refinement (C1 symmetry). The resulting reconstructions shown in Figure 2a, at 7.8 Å resolution (Gold-standard Fourier shell correlation, GS-FSC), correspond to the “open state” crystal structure (PDB 2VMK), yet display significant rearrangements of the large domain and S1 domain (Figure 2b).

One likely explanation could be that the crystal lattice of RNase E favored a particular conformational state, whereas this constraint is absent in the cryo-EM specimen allowing the particles to adopt a more “relaxed” conformation. Another difference between the crystal structure and the cryo-EM map is the symmetry of the tetramer: the crystal structure presents C2 symmetry, but enforcement of that symmetry to the cryo-EM map results in a significant decrease in resolution (from 7.8 Å to 9.5 Å). Map analyses revealed a strong asymmetric pattern in the local resolution distribution (Supplementary Figure S1c). To study the conformational heterogeneity, 3D variability analysis (3DVA) (Punjani et al., 2017; Punjani & Fleet, 2021) was carried out, revealing different modes of molecular motion (Figure 2c). The first variability component represents a synchronous “rocking” movement of two RNase E large domains (LD) (Figure 2c, Component 1). The second mode of variability describes a “rolling” movement of all four large domains (Figure 2c, Component 2), suggesting that the S1 domain can interact with the small domain of a diagonal protomer in an RNase E tetramer (Figure 2c, Component 2, marked with *). The third mode of variability is a “breathing” movement of the tetramer, where it expands and contracts in synchrony between stable conformations (Figure 2c, Component 3). These observations can explain why the final resolution is limited. However, the conformational heterogeneity of the catalytic core is moderate compared to that of the scaffold domains of the degradosome, as will be described in the next sections, and is unlikely to significantly influence ensemble modelling outcomes.

### 2.2 Purification and biophysical analysis of the truncated RNA degradosome

Reproducible protocols were developed for expression and purification of the degradosome and its sub-assemblies to overcome two major challenges, namely the membrane association of the complex and protease sensitivity of the natively unstructured scaffold domain of RNase E. A subassembly of the complex was prepared, referred to here as the “truncated degradosome”, in which the binding site for PNPase has been removed from the C-terminal domain of RNase E, and comprises of RNase E (1-850), RhlB and enolase (Supplementary Figure S2a, Materials and Methods). To prevent the degradation of RNA substrates by RNase E in downstream experiments, a catalytically inactive version of RNase E (1-850) was prepared by the nearly single-atom substitutions in the catalytic site, D303N/D346N.

The oligomeric state, homogeneity, and stability of the degradosome preparations were analyzed by sedimentation velocity analytical ultracentrifugation (AUC) and dynamic light scattering (DLS). For the truncated RNA degradosome, AUC analysis showed that 85% of the particles corresponds to the main peak (Supplementary Figure S2b) with a frictional ratio of 1.48, indicating that the degradosome is homogeneous and extended, and an estimated mass of ~833 kDa, while the theoretical mass for a tetrameric assembly of the truncated degradosome is 945 kDa, which is within the accuracy limits of AUC. DLS measurements confirmed that the truncated degradosome is monodisperse (polydispersity index of 0.173) and extended, with a hydrodynamic size of 330 Å (Supplementary Figure S2c).

### 2.3 SEC-SAXS analyses indicate that the degradosome is highly flexible, but condenses when engaging RNA substrate

To further explore the structural characteristics of the truncated degradosome, size exclusion chromatography coupled to small angle X-ray scattering (SEC-SAXS) experiments were carried out (Mathew et al., 2004; Skou et al., 2014). The purified truncated degradosome was concentrated to micromolar range and resolved by size exclusion chromatography, with direct outflow exposed to the X-ray beam (Materials and Methods). For all samples the truncated degradosome eluted as a single peak, with a constant radius of gyration (*R_g_*) across the profile, indicating monodispersity (Supplementary Figure S3a,b). The scattering intensity curves for the different concentrations were similar in shape (Supplementary Figure S3c) and were merged for further analysis (Franke et al., 2012).

**Figure 3:**
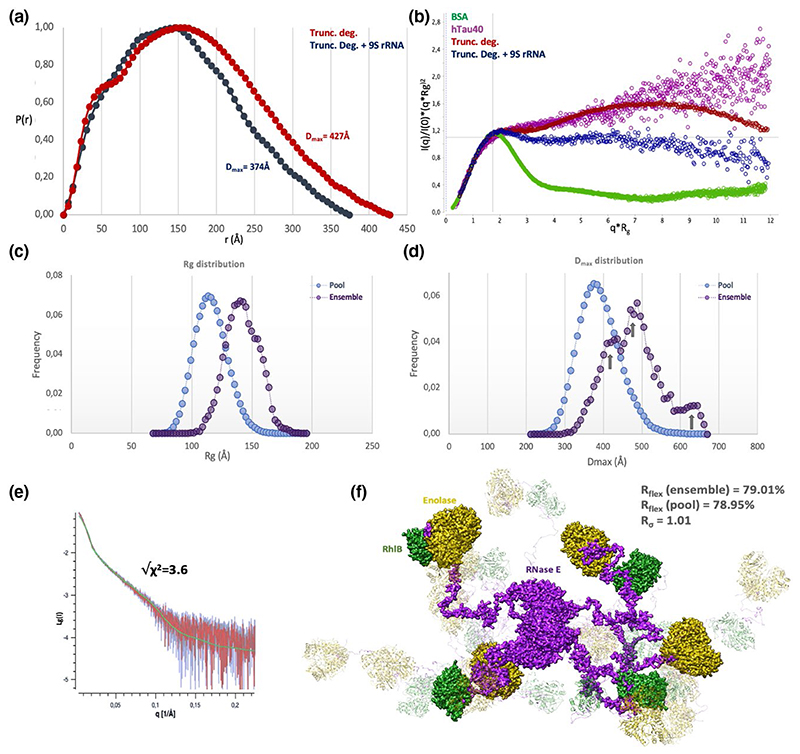
Solution scattering analysis of the truncated degradosome and ensemble optimization modelling. (a) The pairwise distribution function (P(r) function), a distribution function calculated from the SAXS intensity curve that plots all pairwise distances (r, Å) between scattering points within a molecule, extends to a *D*_max_ (the maximum distance within the molecule) of 427 Å for the truncated degradosome (red curve) and a *D*_max_ of 374 Å for the truncated degradosome bound to 9S rRNA (blue curve). The P(r) functions were normalized to unity. (b) Normalized Kratky plots confirm that the complex is more compact in the presence of 9S RNA. A normalized Kratky plot, showing (*q × R_g_)2 × I(q)/I(0*) versus *qR_g_*,with *q* the spatial frequency in 1/Å, *I*(*q*) the scattering intensity at that spatial frequency and *R_g_* the radius of gyration, and these plots allow for qualitative assessment of the flexibility of the complex. The red Kratky curve corresponds to the truncated degradosome alone and the blue Kratky curve to its complex with 9S RNA. For comparison, the green Kratky curve corresponds to BSA (SASDA32), which is folded and compact protein, and the purple curve corresponds to the natively unstructured hTau (Shkumatov et al., 2011). Comparing the truncated degradosome curve with the BSA and hTau Kratky curves, it is clear that the truncated degradosome is a highly flexible protein complex. (c) Distribution of the radii of gyration (Å, x-axis) for the random pool (blue) and the ensembles (purple) of the truncated degradosome. Highly extended truncated degradosome conformers were selected by GAJOE. (d) Distribution of *D*_max_ values (Å, x-axis) for the random pool (blue) and the ensembles (purple) of the truncated degradosome. The different maxima in the ensemble distribution could correspond to distinct clusters of truncated degradosome conformers (grey arrows). (e) Intensity curve of the best ensemble (green) fitted to the experimental SAXS curve (red, sample at 2.2 μM) (i.e., the SAXS scattering intensity Lg(I) as a function of spatial frequency *q*) $\left(\sqrt{\chi^{2}}=3.6\right)$. Experimental error bars are depicted as grey lines. (f) Overlay of the four models in the best ensemble (color coded as in Figure 1b), illustrating the extendedness of the truncated degradosome

The *R_g_* of the truncated degradosome was estimated at 137 Å and the maximum inter-atomic distance (*D*_max_) was estimated at 427 Å, from Guinier analysis and the Pair distribution function (*P_r_*) respectively, suggesting a highly extended shape (Figure 3a, Supplementary Figure S3d). The bimodal shape of the *P_r_* function indicates that the truncated degradosome behaves like a multidomain assembly with flexible linkers, consistent with the extended secondary maximum seen in the normalized Kratky plot (Figure 3a,b). Interestingly, SEC-SAXS analysis of the truncated degradosome bound to 9S RNA, a highly structured RNase E substrate and a precursor of the 5S ribosomal RNA (Ghora & Apirion, 1978), reveals a more compacted assembly, with a *R_g_* of 123 Å and a *D*_max_ of 374 Å (Figure 3a, Supplementary Figure S3c,d). The Pairwise distribution function for the truncated degradosome bound to 9S rRNA is less bimodal in shape. Moreover, the normalized Kratky plot shows a less pronounced secondary maximum for the complex (Figure 3b). Taken together, these results indicate that the truncated degradosome compacts upon binding RNA substrates.

### 2.4 Ensemble modelling sheds light on the conformational landscape of the truncated degradosome

The flexibility of the degradosome limits the information that can be inferred from standard SEC-SAXS analyses and prevents ab initio shape reconstructions, which would only reflect an average over all existing conformations. To evaluate the inherent conformational heterogeneity of the degradosome, we used an adapted ensemble optimization modelling (EOM) approach (Bernado et al., 2007; Tria et al., 2015). EOM explores the conformational landscapes of proteins in solution based on a priori structural information and the experimental scattering intensity to find a population of structures that best fits the experimental scattering data.

Crystal structures were used as a priori structural information for the tetrameric RNase E catalytic domain in apo-form (Koslover et al., 2008, pdb 2VMK) and enolase bound to a fragment of the RNase E scaffold domain (Nurmohamed et al., 2010, pdb 3H8A). No experiment-based structure is available for *E. coli* RhlB, but a homology model based on VASA helicase from *Drosophila* has been generated in prior studies (Bruce et al., 2018; Sengoku et al., 2006). CABS-Dock (Blaszczyk et al., 2016) was used to model the interaction between RhlB and the corresponding binding fragment of RNase E (as found by Chandran et al., 2007) with constraints based on hydrogen-deuterium exchange mass spectrometry experiments (Bruce et al., 2018). As such, the RNase E scaffold domains are the only parts of the truncated degradosome that are unaccounted for, and therefore will be sampled via EOM (Supplementary Figure S4a).

A tailored EOM pipeline was developed, in which models of the truncated degradosome protomer were generated with RANCH (Bernado et al., 2007) by treating the C-terminal RNase E scaffold domain as “random coil”, creating four “monomer” pools, one for each RNase E component in the truncated degradosome (Supplementary Figure S4b). Starting from these four pools, random combinations were selected to generate a “tetramer pool” of candidate truncated degradosome structures (Supplementary Figure S4c) and in silico intensity profiles calculated for each member of the pool (Supplementary Figure S4d; Svergun et al., 1995) as input for a genetic algorithm (GAJOE) that selects ensembles of structures from the random pool that fit the experimental scattering profile (Supplementary Figure S4e). The resulting distribution functions of the *R_g_* and *D*_max_ metrics for selected ensembles were plotted and compared to the corresponding distribution functions for the random pool (Figure 3c,d). These distribution functions show that the ensemble models are significantly more extended than the overall pool, meaning that the RNase E scaffold domain and its partner enzymes extend away from the catalytic core of RNase E in solution, ruling out a closed configuration of the degradosome components in the absence of RNA.

The observed flexibility of the truncated degradosome is also indicated by quantitative metrics of the size distributions (Tria et al., 2015). R_flex_ is an entropy-based estimation of the flexibility of a pool of models, ranging from complete rigidity (0%) to maximal flexibility (100%). For the truncated degradosome ensemble, the *R*_flex_(ensemble) = 79.01%, and for the random pool *R*_flex_(pool) = 78.95%. The variance *R*_σ_ is a measure of how wide a range of model sizes are populated by the ensembles compared to the random pool. The calculated *R*_σ_ was 1.01, meaning that the model sizes in the ensembles have as much variation as those in the random pool (but shifted to significantly higher values). Both *R*_σ_ and *R*_flex_ indicate that the ensemble modelling could not identify a set of rigid truncated degradosome models to explain the SAXS data, because many highly extended conformations are adopted in solution (Figure 3c-f). The best fitting ensemble ($\sqrt{\chi^{2}}=3.6$, Figure 3e) consists of four extended truncated degradosome models and is depicted in Figure 3f. The *D*_max_ distribution of the ensembles has three maxima, which suggests that clusters of more extended or compact conformers could exist in solution.

### 2.5 Cryo-electron tomography of the membrane bound RNA degradosome

The *E. coli* RNA degradosome is anchored to the inner membrane through a microdomain in the RNase E C-terminal scaffold domain, and membrane association could potentially induce the assembly to adapt a more defined conformation (Figure 1b). The truncated degradosome was reconstituted onto vesicles with a lipid mixture composed of 70% (w/w) 1,2-dioleoyl-sn-glycero-3-phosphoethanol amine (DOPE) and 30% (w/w) 1,2-dioleoyl-sn-glycero-3-phospho-(1 ‘-rac-glycerol) (DOPG) to mimic the *E. coli* plasma membrane. While DOPE is neutral at physiological pH, DOPG is negatively charged. It is important to note that the RNase E membrane attachment helix is amphipathic, with one side of the helix consisting of non-polar amino acids, which embed into the lipid membrane. The other half is positively charged at neutral pH and has been proposed to play a role in facilitating initial interactions with the negatively charged DOPE/DOPG-based *E*. *coli* membrane (Strahl et al., 2015). The truncated degradosome (solubilized in the detergent beta-DDM) and liposomes were mixed and dialyzed overnight in a detergent-free buffer, to favor reconstitution upon removal of detergent in the sample. Subsequent ultracentrifugation assays in a 10%–40% continuous glycerol gradient indicated that membrane association of RNase E was highly efficient (Supplementary Figure S5).

**Figure 4:**
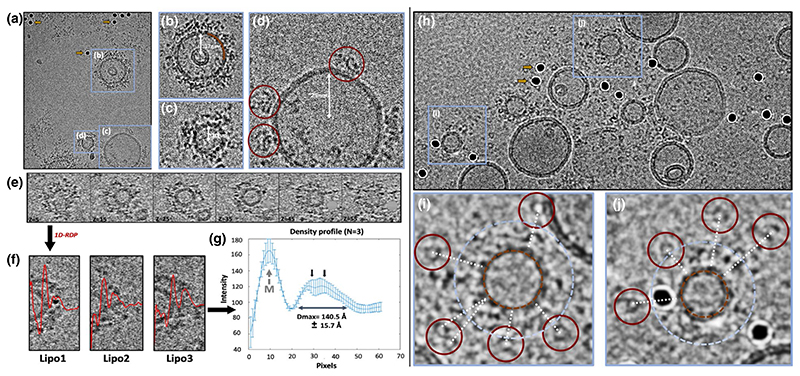
Reconstitution, vitrification, and imaging of membrane bound degradosome assemblies. (a) A representative cryo-EM image of the degradosome-coated liposomes (blue boxes) (3 μm defocus). Gold fiducials are highlighted with yellow arrows. (b–d) Close ups of selected lipid vesicles. In panel b the lipid membrane is highlighted with a brown curve. In panel (d) individual membrane associated degradosome particles are highlighted with red circles. White arrows and labels show the estimated radius of the lipid vesicles. (e) Slices through a selected liposome along the Z-axis. Values for Z in each slice correspond to the frame number. The grey sphere at the bottom right of the insets corresponds to a gold fiducial that was replaced by random noise. (f) 1-dimensional radial density profiles (1D-RDP, red) for three different liposomes. (g) Averaged 1D-RDP for the three liposomes depicted in (e). The membrane peak is annotated (M). The extension of the membrane bound truncated degradosome was estimated at 140.5 (±15.7) Å. Black arrows indicate the putative bimodal shape of the radial density profile. (h) Representative image of the liposome-bound full-length RNA degradosome (3 μm defocus). Yellow arrows point to two gold fiducials. (i,j) PNPase (red circles) is located further away from the lipid membrane (brown circle) than the average extendedness of the truncated degradosome (light blue circle)

Cryo-EM images of the specimens revealed that most of the liposomes were densely packed with protein (Figure 4a–e). A few liposomes, however, carried distinguishable particles of around 20 nm (Figure 4d). Although liposomes were prepared by extrusion with a 50 nm porous membrane, they are heterogeneous in size, ranging from 10 to 70 nm in diameter, and smaller liposomes were more densely packed with protein than larger liposomes. Decreasing the ratio of truncated degradosome: liposome in the sample resulted in more empty liposomes, rather than reducing the overall degradosome packing on the liposomes (data not shown).

Tilt series were collected for a vitrified liposome-degradosome sample and liposomes enriched in protein were identified in the resulting tomograms by visual inspection and selected for further analysis (Figure 4e,f). The 1-dimensional radial density profile (1D-RDP) was calculated from the center outwards for each liposome (Figure 4f) and an averaged curve was generated (Figure 4g). Tracing the density from the middle of the liposome outwards, the first big peak corresponds to the lipid membrane (Figure 4g, grey arrow). Interestingly, for every liposome there is a gap of ~30 Å between the density of the liposome and the density of the truncated degradosome. The density peak for the truncated degradosome itself is relatively compact and extends 140.5 (±15.7) Å away from the membrane (Figure 4g). This measure of the extension can be regarded as the real space equivalent of *D*_max_ in SEC-SAXS (Figure 3a). In solution, however, the *D*_max_ of the truncated degradosome was measured at 427 Å based on the P(r) function. Due to the surface barrier of the membrane, the extension as measured by the 1D-RDP should be compared to half the *D*_max_ in solution, that is, 213.5 Å. Regardless, the extension of the truncated degradosome bound to a membrane (140.5 Å) is significantly lower than in solution (213.5 Å), suggesting that the truncated degradosome is more compact when associated to the membrane. It should be noted that the extension estimate from the 1D-RDP only represents the radial distribution of the truncated degradosome along the normal vector to the membrane surface. Thus, the discrepancy between the 1D-RDP and the *D*_max_ in solution could arise also from lateral spreading of the degradosome over the membrane, much like a flower blooming.

The density profile was often bimodal (Figure 4f,g), which could hint towards a higher level of structural organization of the different components of the degradosome on the lipid membrane. To study how the different components of the degradosome could be spatially organized, the full RNA degradosome was reconstituted on lipid vesicles and vitrified. Cryo-EM images of the full RNA degradosome bound to liposomes show that the majority of PNPase is localized in liposome-rich regions on the grid (Figure 4h–j). PNPase particles can be recognized as ring-like particles in the vitreous ice layer. Interestingly, the PNPase particles are situated further away from the membrane than the measured extent of the truncated degradosome (Figure 4i–j). The full-length RNase E has 200 more amino acids than the truncated degradosome, and the binding site for PNPase is localized at the very end of its scaffold domain (Figure 1). The location of PNPase at a distance from the surface of the lipid vesicles suggests that PNPase may hover flexibly on the periphery of the membrane-bound degradosome assembly in the cell.

### 2.6 Structural studies of putative surveillance assemblies

Previous studies have proved that the bacterial RNA degradosome can interact directly with translating ribosomes, introducing the possibility of putative surveillance assemblies (Hamouche et al., 2021; Leroy et al., 2002; Redko et al., 2013; Tsai et al., 2012). The binding of the 70S ribosome and the 30S ribosomal subunit to the membrane-associated truncated degradosome was tested by ultracentrifugation assays in glycerol gradients (Supplementary Figure S6). As expected, the 30S small ribosomal subunit did not interact with lipid membranes, as the 30S bands did not shift along the gradient in the presence of liposomes (Supplementary Figure S6a,b). However, when the truncated degradosome was reconstituted on the liposomes, there was a significant shift of 30S in the glycerol gradient, which co-sedimented with the truncated degradosome (Supplementary Figure S6c). The 70S ribosome was also found to co-sediment with the truncated degradosome-liposomes (Supplementary Figure S6d).

**Figure 5:**
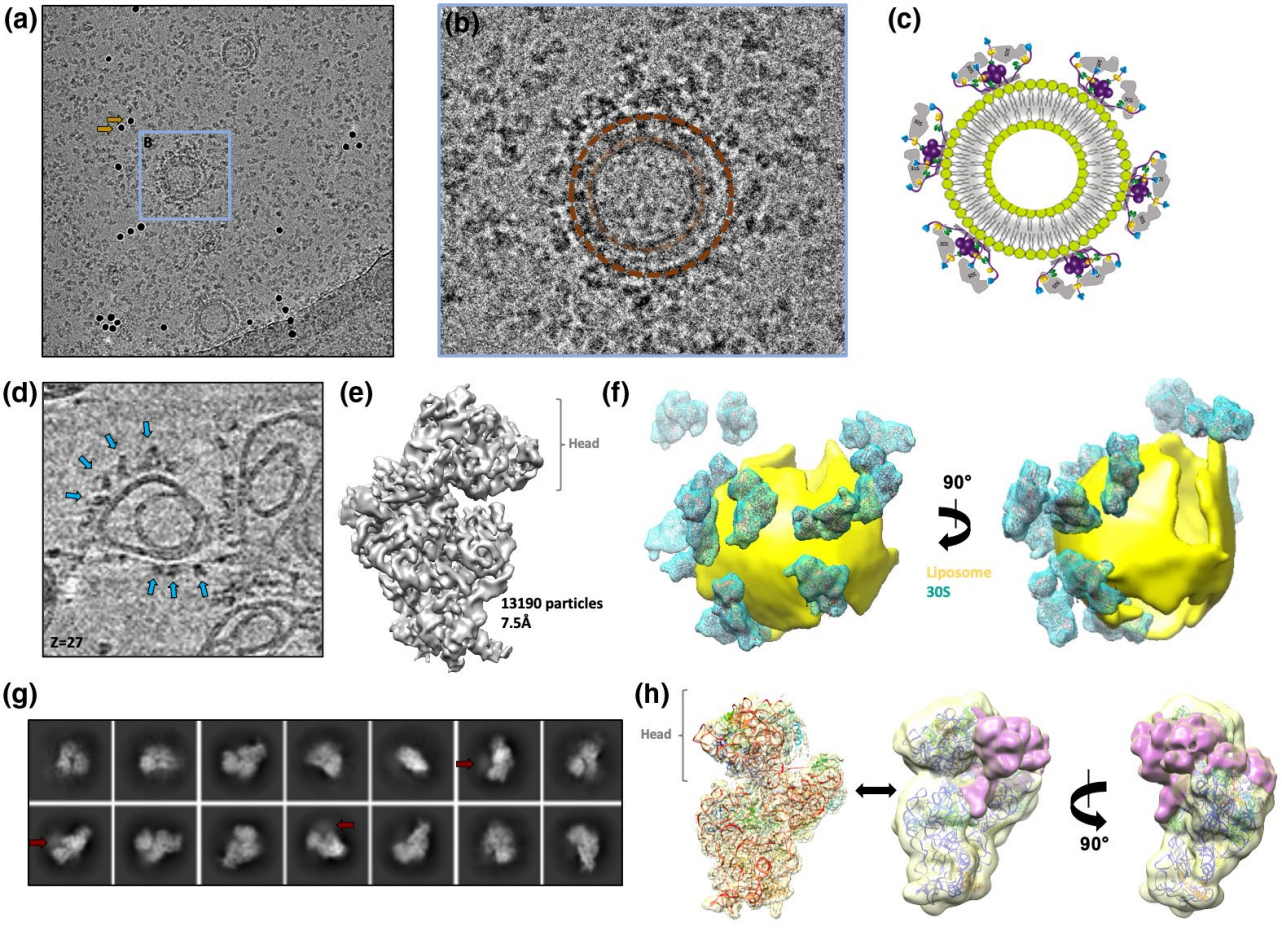
Cryo-EM studies on 30S super-complexes. (a) Representative cryo-EM image of truncated degradosome-coated liposomes and 30S small ribosomal subunits (3 μm defocus). Two gold fiducials are highlighted by yellow arrows. (b) Magnified representation of a liposome (modelled with a dark brown circle), which coincidentally encloses a second liposome (light brown circle). 30S particles are enriched on the outer liposome, which is saturated with truncated degradosome. Adapted from Bandyra et al. (2012). (c) Schematic representation of a lipid vesicle coated with degradosome-30S super-complexes. (d) Magnified image of a slice through a reconstructed tomogram showing distinct 30S particles near the lipid membrane of a liposome (blue arrows). (e) Sub-tomogram average as reconstructed with Relion and M. 13,190 sub-tomogram volumes contributed to this average, which has an estimated resolution of 7.5 Å (GS-FSC). (f) The raw, noisy tomograms (top) were processed with TomoSegMemTV to model the liposome membranes (middle, membranes are modelled in yellow). A spatial distribution volume of the 30S particles was generated by mapping back the reconstructed averages, lowpass filtered to 15 Å to replace the original, noisy 30S particles in the tomogram (30S averages are in cyan). (g,h) 2D class averages and 3D map for the recognition core bound to the 30S small ribosomal subunit. 2D class averages reveal diffuse additional densities near the 30S head region (red arrows). 3D reconstructions of the super-complex and comparison to a cryo-EM map of 30S alone reveal weak extra densities spanning across the mRNA exit site (purple)

Cryo-electron tomography was used to study the interaction between 30S small ribosomal subunit and the membrane-bound truncated degradosome (Figure 5a). A subset of the 30S particles is enriched at the membrane-bound truncated degradosome (Figure 5b,c). After collecting tilt series and reconstructing tomograms for these samples, it was apparent that free floating 30S particles aligned to the air-water interfaces of the vitreous ice (data not shown). 30S particles associated with the membrane associated truncated degradosome, are, on the other hand, pulled away from the air-water interface (Figure 5d, blue arrows). After aligning and averaging 30S sub-tomogram volumes (see Materials and Methods), a consensus map of the 30S ribosome was reconstructed at 7.5 Å resolution (GS-FSC), shown in Figure 5e. No additional density could be observed for the truncated degradosome on the 30S map. Since the majority of ribosomes are not bound to the truncated degradosome and aligned to the air-water interface, it is likely that additional density corresponding to the truncated degradosome was averaged out during the alignments due to low occupancy. To better visualize the 30S particles associated with the truncated degradosome, the reconstructed 30S average was mapped back into the raw tomograms, conserving their original position and orientation (Figure 5f). As noted earlier, most ribosomes are at the air-water interface and therefore are at the top and at the bottom peripheries of the tomographic volume. Subsequently, lipid vesicles were traced and modelled (Figure 5f), and the resulting reconstructions displayed several 30S small ribosomal subunit particles in close proximity to the liposomes.

The ultracentrifugation and cryo-ET studies presented above show that the 30S small ribosomal subunit colocalizes with the membrane-associated RNA degradosome, but the 30S averages did not resolve any density for the RNA degradosome. Previous studies showed that the interaction between the RNA degradosome and ribosomes is mediated mainly by RhlB and the two RNA binding sites on the RNase E scaffold domain (RBD and AR2, Figure 1b; Tsai et al., 2012). A smaller sub-assembly of the RNA degradosome, referred to as the recognition core, was used to structurally elucidate this binding. The recognition core is comprised of a short fragment of the flexible RNase E scaffold domain (RNase E 603-850) carrying the two RNA binding domains and the binding sites for RhlB and enolase, but not the membrane attachment helix (Bruce et al., 2018). Electrophoretic mobility shift assays showed that the recognition core forms a super-complex with the 30S small ribosomal subunit (Supplementary Figure S7a) and this super complex was co-purified by size exclusion chromatography for cryo-EM studies (Supplementary Figure S7b). Two small datasets were collected, one for the 30S-recognition core super-complex and one for 30S alone, as a negative control. For the 30S-recognition core sample, 2D class averages show diffuse additional densities near the 30S head (Figure 5g) and subsequent 3D reconstructions revealed weak extra density near the 30S head/exit channel, despite the limited resolution (Figure 5h). The extra density was not observed in the negative control (30S alone), so it is likely to correspond to the recognition core. The low resolution of the recognition core can be explained by its significant conformational heterogeneity, which is not fully constrained by the 30S small ribosomal subunit. Attempts at reliable docking models for enolase and RhlB were unsuccessful.

## 3 Discussion

Over the past 25 years, many studies have investigated the functional roles of bacterial RNA degradosome assemblies in post transcriptional gene regulation (Bandyra & Luisi, 2018; Bayas et al., 2018; Chao et al., 2017; Commichau et al., 2009; Tejada-Arranz et al., 2020). Although many of its components have been structurally elucidated, the structure of the RNA degradosome in its entirety is still elusive. The main challenge for structural studies is the flexibility of the disordered scaffold domain of RNase E. Previous crystallographic studies showed that the catalytic domain of RNase E adopts different conformational states in the absence of RNA substrates (Bandyra et al., 2018; Callaghan et al., 2005; Koslover et al., 2008), and our cryoEM results corroborate this conformational flexibility (Figure 2b,c). In the absence of RNA, three principal modes of conformational freedom were resolved (Figure 2c). It is likely that this inherent flexibility in the RNase E core adds only a small contribution to the overall flexibility of the RNA degradosome due to the natively unstructured character of the RNase E scaffold domain. SEC-SAXS analyses of the truncated degradosome provide the first description of its conformational behavior in solution and indicate an extended complex emanating flexibly from a tetrameric core. The results are in line with previous studies indicating conformational heterogeneity of the RNase E scaffold domain (Bruce et al., 2018). An ensemble approach, EOM, confirmed that the truncated degradosome adopts highly extended conformations in solution (Figure 3f). Interestingly, the binding of 9S, an RNA substrate of the degradosome, causes compaction of the complex.

To mimic the cellular environment of the degradosome more closely, we reconstituted the degradosome onto small lipid vesicles. As these have a high degree of positive curvature, they do not fully mimic the gentle negative curvature of the cytoplasmic membrane in the cell. Nevertheless, the difference in curvature is not expected to have a profound impact on the local interactions of adjacent degradosomes. The extension of the truncated degradosome when bound to lipid membranes, evaluated by 1-dimensional radial density profiles (1D-RDP), shows that it is more compact compared to its state in solution. Moreover, the 1D-RDP suggests that the main body of the truncated degradosome is separated from the membrane (Figure 4g). Cryo-EM images of the whole RNA degradosome reconstituted on liposomes suggest that PNPase mainly resides at the periphery of the membrane-bound protein density (Figure 4h-j). This too points towards a degree of organization of degradosome components in relation to the membrane. As the RNA degradosome works as an integrated molecular machine in the cell, digesting RNA substrates down to individual ribonucleosides, some organization within its inherent conformational chaos is likely to be necessary to facilitate activity and ensure completion of the RNA degradation pathway.

Sub-tomogram averaging on the membrane-bound RNA degradosome bound to 30S ribosomal subunits and subsequent mapping of the averages in the original tomogram revealed that the 30S subunit can bind the membrane-associated truncated degradosome (Figure 5e,f). 3D classification of sub-tomogram volumes did not satisfactorily resolve large entities of extra density such as the truncated degradosome or lipid membranes and other classification tools may be required to address this in the future. Cryo-EM SPA of the degradosome recognition core bound to the 30S subunit reveals weak cryo-EM density near the head of 30S and provides the first visualization of the RNA degradosome scaffold region and its participation in a putative surveillance assembly. In addition to interactions with the 70S ribosome (Tsai et al., 2012), it is possible that the degradosome interacts with 30S-based translation initiation assemblies such as the 30S pre-initiation complex (PIC) in vivo as a fidelity check (Hussain et al., 2016). The latter is an assembly in which three initiation factors help pre-organize the start codon of a mRNA at the P-site of the 30S ribosomal subunit and enable selecting initiator tRNA before full ribosome assembly and translation elongation (Milón & Rodnina, 2012).

The degradosome is highly polarized for charge distribution. Table 1 provides calculated isoelectric points for the degradosome components estimated using ProtParam. Like many intrinsically disordered proteins, the scaffold domain of the RNA degradosome is enriched in polar and charged residues and can be defined as a polyampholyte (Das & Pappu, 2013). While most segments are electronegative, there are strongly basic portions in the C-terminal tail of RhlB and in the RNA binding segments and recruitment sites for enolase and RhlB of RNase E (603-850). The estimated κ-values calculated with CIDER (Holehouse et al., 2015) for the electropositive region (603-850) and for the electronegative PNPase interacting site (851-1061) differ (respectively 0.199 and 0.149) and point towards a distinct structural organization of these two regions: the negative portion of the scaffold domain corresponds to a weak polyampholyte, while the electropositive region corresponds to a strong polyampholyte (with FCR = 0.395), that could fold into an hairpin-like conformation in the absence of bound RNA. This electropositive region is expected to be clustered spatially, and, in the absence of bound RNA, to have favorable interactions with the negatively charged head groups of the membrane (Figure 1b). Binding with RNA is expected to impact on the potential interaction of this portion of the degradosome with the membrane, possibly resulting in its displacement radially from the membrane surface, as observed in Figure 4f and g. The change in the charge distribution subsequent to the binding of RNA is likely to impact on the structural organization of the RNA degradosome, in the manner of an “electrostatic switch” causing part of the body of the complex to extend away from the membrane, and on the extendedness of the scaffold domain, causing its compaction. This is in accord with the findings of compaction of the assembly in solution by SEC-SAXS. These two actions together may facilitate the interaction between the different components bound to the scaffold domain of the RNA degradosome in the presence of an RNA substrate.

Perhaps one reason for the extraordinary conservation of the intrinsically disordered characteristic region of the scaffold domain is that it might drive the RNA degradosome into microscopic condensate in the presence of RNA, with functional consequence (Aït-Bara & Carpousis, 2015; Al-Husini et al., 2018). Recent studies showed how the RNA degradosome from *C. crescentus*, like many other RNA binding proteins, can undergo LLPS depending on the presence of RNA (Al-Husini et al., 2018, 2020). LLPS behavior is a macroscopic property and underpinned by highly dynamic microscopic processes (Schmit et al., 2021). The macroscopic organization is a condensate that is on the order of a fraction of a micron, and the boundary is set by the balance of forces of surface tension of the droplet and the free energy driving molecular crowding within the droplet. The microscopic origins of the effect can be rationalized by models of colloidal polymers, where molecular concentration is driven by self-association balanced by repulsion (Schmit et al., 2021). As the degradosome is highly acidic, except at the RNA binding domains in region 603-850, interactions with the electronegative lipid surface are expected to provide an electrostatic force that directs the body of the polymer away from the membrane along the normal to the membrane plane, but in the opposite direction for the RNA binding domains (Figure 1b and Table 1). This prediction is in line with the observation of PNPase trimers, which associate with the very C-terminus of the RNase E scaffold domain at the periphery of membrane bound RNA degradosome density in our cryo-EM images (Figure 4h–j). In addition, the apparent gap between the bulk truncated degradosome density and the lipid membrane, as calculated from the 1D RDPs, further supports this (Figure 4g). Engagement of the RNA binding domains with the negatively charged RNA polymer will change the charge distribution, with a net force directing the degradosome further away from the membrane. Furthermore, binding nucleic acid might enable shared interactions with the RNA between adjacent degradosome assemblies.

Thus, we envisage that RNA binding to the RNA degradosome might alter its relationship to the membrane surface and enable association of neighboring degradosomes through shared binding of the nucleic acid. This “electrostatic switch” may also present more nonpolar residues that would have a dehydrating effect locally, and drive molecular crowding that is expected to be balanced by charge repulsion. Local clustering of the degradosome, observed in vivo, is expected to be transient and unstable state from Brownian encounters of the concentrates. Our tomographic reconstructions of the membrane bound truncated degradosome display its tendency for such clustering events even in the absence of RNA, as some of the lipid membranes were densely packed with protein, while other liposomes were free of bound degradosome.

## 4 Material and Methods

### 4.1 RNase E catalytic domain expression and purification

BL21(DE3) cells harboring the plasmid pRne529-N, which encodes RNase E catalytic domain with N-terminal 6xHis-tag, were grown in 2×YT media (Formedium) supplemented with 100 μg/ml carbenicillin at 37°C. At OD_600_ = 0.6, the cultures were induced with 1 mM IPTG, cells were harvested by centrifugation after 3 hr of incubation at 37°C. The cell pellets were then resuspended in buffer A (20 mM Tris-HCl pH 7.9, 500 mM NaCl, 5 mM imidazole, protease inhibitor cocktail tablet [Roche]), supplemented with DNase I (1 μg/ml), and passed through an EmulsiFlex-05 cell disruptor (Avestin) at 10–15 kbar for cell lysis. The lysate was clarified by centrifugation (4°C, 30 min, 37,500×*g*) and loaded on a 5 ml HiTrap Chelating HP column (GE Healthcare) charged with NiSO_4_. Proteins were eluted by an imidazole gradient (buffer A supplemented with 1 M imidazole) and evaluated by SDS–PAGE. Fractions enriched in RNase E catalytic domain were pooled, concentrated to 2 ml with 15 ml Amicon Ultra 30,000 MWCO concentrator (Millipore) and loaded on to a Sephadex 200 gel filtration column (GE Healthcare) equilibrated with buffer containing 20 mM Tris pH 7.9, 500 mM NaCl, 10 mM MgCl_2_, 0.5 mM EDTA, 1 mM TCEP, 5% vol/vol glycerol and a protease inhibitor cocktail tablet (Roche). Eluted fractions were analyzed by SDS–PAGE and those containing purified RNase E catalytic domain were flash frozen with liquid nitrogen and stored at –80°C. The protein concentration was determined spectroscopically using a NanoDrop ND-1000 spectrophotometer (Thermo Scientific) and a *λ*_280nm_ extinction coefficient of 29,005 M^–1^ cm^–1^ per RNase E (1-529) monomer.

### 4.2 Expression and purification of the recognition core

Expression and purification of the recognition core were carried out following standard protocols described in Bruce et al. (2018).

### 4.3 Preparation of RNA degradosome and truncated degradosome constructs

Expression plasmids for wild-type RNase E (1-850) and different RNase E (1-529) mutants were available in the lab. Primers for PCR reactions were designed using PrimerX (www.bioinformatics.org/primerx/; Table 2) and amplification was performed with Pfu Turbo polymerase (NEB). All mutations were incorporated from existing RNase E (1-529) constructs and verified by sequencing.

Two mutations were introduced in the RNase E catalytic domain, D303N/D346N and D346C. To do this, a fusion-PCR strategy was used, comprising of two successive PCR reactions. In the first step, two joining fragments of RNase E (1-850) were PCR-amplified, that is, RNase E (1-445) and RNase E (445-850), with complementary overhangs. For both mutations, the first RNase E fragment (1-445) was amplified from an existing construct of RNase E (1-529) containing these mutations (provided by Dr. Katarzyna Bandyra and Dr. AJ Carpousis, respectively). The second RNase E fragment (1-850) was amplified from a wild-type construct of RNase E (1-850) (provided by Dr Katarzyna Bandyra). PCR products were resolved on a 1% low melting point agarose gel (Sigma), the bands of interest were excised and, after melting the matrix by incubation at 70°C, mixed in a single second PCR reaction which amplified the whole RNase E (1-850) gene, containing the desired mutations. The product of the last PCR was digested with SalI and EcoRI (NEB) and resolved on a 1% low melting point agarose gel. The gel band was excised and directly ligated with T4 ligase (NEB) into a pRSFDuet plasmid containing the RhlB gene in one of its multiple cloning sites. The latter had been digested with the same restriction enzymes and dephosphorylated with CIP (NEB) according to the manufacturer’s instructions. The primers used for the PCR reactions are listed in Table 2.

One inactivating double mutation, D303N/D346N, was introduced in the catalytic domain of the full-length RNase E (1-1064), following the same protocol. The first fragment was amplified from the same existing RNase E (1-529) construct bearing the D303N/D346N mutations. The second fragment was amplified from a wild-type RNase E (1-1064) construct available in the lab (provided by Dr. AJ Carpousis). The primers used are listed in Table 3. After fusion PCR, the new construct was cloned in a pRSF plasmid via In-Fusion cloning. To generate complementary ends between the pRSF plasmid and the insert and to linearize the plasmid, PCR reactions were used. Primers are listed in Table 3. Plasmid and insert were resolved on 1% agarose gel (Sigma-Aldrich), the bands of interest were excised and extracted with QIAquick Gel Extraction Kit (QIAGEN). The In-Fusion reaction (Takara Bio) was performed according to manufacturer’s instructions.

### 4.4 In vivo reconstitution of the truncated degradosome

Building on the protocol of Worrall et al. (2008), *E. coli* ENS134-10_eno cells (carrying the pET21b_eno plasmid encoding enolase) were transformed with pRSF-DUET_rne1-850/rhlb, encoding 6xHis-tagged RNase E (1-850) and RhlB, and used to grow cultures in 2×YT (Formedium) at 37°C supplemented with 15 μg/ml kanamycin and 25 μg/ml carbenicillin. When OD_600_ reached 0.3–0.5, expression was induced with addition of 1mM IPTG and cultures were incubated overnight at 18°C. Cells were harvested by centrifugation with a Beckman JS 4.2 rotor at 5,018 rcf and resuspended in NiA buffer (50 mM Tris-HCl pH 7.5, 1 M NaCl, 100 mM KCl, 10 mM MgCl_2_, 1 mM TCEP, 0.02% (w/v) β-DDM (β-dodecylmaltoside), supplemented with a complete EDTA-free protease inhibitor tablet (Roche). 1% Triton X-100 was added to help solubilize membrane associated proteins during lysis, along with 1 mM TCEP, 1 mM PMSF and 2 μg/ml of DNase I and 20 mg/ml of β-DDM. After lysis by cell rupture (Emusliflex-05, Avestin, 5 passes 10–15 kbar), the lysate was centrifuged at 4°C (38,000×g, 30 min) and loaded on a 5 ml HiTrap chelating HP column (GE healthcare), freshly charged with NiSO_4_ and equilibrated with NiA buffer. The truncated degradosome was eluted with a linear 0%–60% NiB gradient (NiA +500 mM imidazole). Protein containing fractions were analyzed with SDS PAGE, pooled and diluted (1:3) with SP C buffer (50 mM Tris-HCl pH 7.5, 0.02% w/v β-DDM) before loading on a 1 ml HiTrap SP HP column (GE Healthcare) equilibrated with SP A buffer (50 mM Tris-HCl pH 7.5, 50 mM NaCl, 10 mM KCl, 1 mM TCEP 0.02% w/v β-DDM). The truncated degradosome was eluted with a linear gradient (0%-50%) of SP B buffer (50 mM Tris-HCl pH 7.5, 2 M NaCl, 10 mM KCl, 1 mM TCEP, 0.02% (w/v) β-DDM). The protein-containing fractions were pooled and concentrated with 100 kDa cutoff Amicon Ultra centrifugation filter (Millipore) according to the manufacturer’s protocol. The concentrated sample was then loaded on a Superose 6 10/300 GL size-exclusion column (GE Healthcare) equilibrated with S6 running buffer (50 mM HEPES pH 7.5, 400 mM NaCl, 100 mM KCl, 1 mM TCEP, 0.02% (w/v) β-DDM). Peak fractions were analyzed with SDS PAGE and clean fractions were supplemented with 10% v/v glycerol before storage at –80°C. For subsequent experiments, protein concentrations were determined spectroscopically using a NanoDrop ND-1000 spectrophotometer (Thermo Fisher Scientific) and a *λ*_280nm_ extinction coefficient of 119,990 M^–1^ cm^–1^ for a protomeric truncated degradosome unit.

### 4.5 In vitro reconstitution of the RNA degradosome

Several approaches were taken to purify the whole *E. coli* RNA degradosome, but the yield was low. Therefore, an in vitro reconstitution protocol for the full RNA degradosome was developed. Enolase, PNPase and RhlB were expressed separately.

*E. coli* C43 cells were transformed with pET15_wtrne encoding 6xHis-tagged wild-type full-length RNase E, or pET15_NNrne encoding the D304N/D346N inactive full-length RNase E. Cells were grown at 37°C in 2×YT media (Formedium) supplemented with 50 μg/ml kanamycin. At OD_600_ = 0.6, the cultures were induced with 1 mM IPTG and incubated overnight at 16°C. Untagged Enolase (pET21b_eno), PNPase (pET-Duet_pnp) and RhlB (pRSF_Duet_rhlb) were expressed separately in *E. coli* BL21(DE3) cells according to standard protocol (1 mM IPTG induction at OD_600_ = 0.5, 3–4 hr of expression at 37°C). All cells were mixed together before lysis, corresponding to 6 × 0.5l of RNase E cultures, 1 × 0.5l of PNPase culture, 1 × 0.5l of enolase culture and 1 × 0.5l of RhlB culture. The protein concentration was determined spectroscopically using a NanoDrop ND-1000 spectrophotometer (Thermo Scientific) and a *λ*_280nm_ extinction coefficient of 218,090 M^–1^ cm^–1^ per protomeric RNA degradosome unit.

### 4.6 Sedimentation velocity analytical ultracentrifugation (SV-AUC)

SV-AUC (Cole et al., 2008) was performed using a Beckman Optima XL-I analytical ultracentrifuge (Biophysics facility, Department of Biochemistry, University of Cambridge). Absorbance data was collected at a wavelength of 280 nm for the truncated degradosome sample, which was concentrated to a final concentration of 4 mg/ml in buffer S6 (50 mM Tris-HCl pH 7.5, 400 mM NaCl, 100 mM KCl, 5 mM MgCl_2_, 1 mM TCEP, 0.02% β-DDM, 10% (v/v) Glycerol). The samples were spun at 4°C, 50,000 rcf for 12 hrs. The obtained data were analyzed by the Sedphat program and modelled using Sedfit software (Zhao et al., 2013). Buffer viscosity was calculated with SEDNTERP (http://jphilo.mailway.com).

### 4.7 Dynamic light scattering (DLS)

Truncated degradosome samples were diluted in S6 buffer (50 mM Tris-HCl pH 7.5, 400 mM NaCl, 100 mM KCl, 5 mM MgCl_2_, 1 mM TCEP, 0.02% β-DDM, 10% (v/v) glycerol) to final concentration of 0.5 mg/ml. A buffer condition with a lower salt content was also tested for signs of protein aggregation (50 mM Tris-HCl pH 7.5, 100 mM NaCl, 150 mM KCl, 5 mM MgCl_2_, 1 mM TCEP, 0.02% β-DDM). For the latter, buffer exchange was carried out with a PD MiniTrap G-10 column (GE Healthcare) column. A final sample volume of 70 μl was subjected to DLS measurements using a *Zetasizer Nano S* (Malvern Panalytical). For each experiment three technical replicates, with 15 runs each, were collected.

### 4.8 Small angle X-ray scattering coupled to size exclusion chromatography (SEC-SAXS)

The samples were concentrated, flash frozen in liquid nitrogen and sent to beamline B21 at Diamond Lightsource (Harwell campus, Didcot, United Kingdom) for data collection. The sample was loaded on a Superose 6 Increase 3.2/300 column (GE Healthcare) by a High-Performance Liquid Chromatography (HPLC) instrument (Agilent) directly before elution into the sample detection chamber, where a monochromatic beam illuminated the sample as it flowed through. 2D scattering profiles were radially averaged on the fly at the beamline. For each sample the Radius of Gyration (*R_g_*) was plotted as a function of the elution frame for optimal selection of a homogeneous sample range in Scatter (biosis.net). Temporal averaging of the selected range and buffer subtraction were carried out in Scatter and in DataSW to generate the final Intensity curve (biosis.net, Shkumatov & Strelkov, 2015). Further analysis of the Intensity curves was performed in Scatter. ALMERGE (Franke et al., 2012) was used to merge intensity curve from different concentrations.

For flexibility assessment of the truncated degradosome, the EOM approach was used (Bernado et al., 2007; Tria et al., 2015) with a tailored pipeline, in which models of the truncated degradosome protomer were generated with RANCH (Bernado et al., 2007) and FULCHER (Shkumatov et al, in preparation; converts the models to all-atom models) by treating the RNase E scaffold domain as “random coil” and subsequently filtered to remove models with MOLPROBITY clash score worse than 60. This generated four “monomer” pools, one for each RNase E component in the truncated degradosome, of 20,000 protomers each (Supplementary Figure S4b). To overcome the single chain restriction, RhlB and enolase were made “invisible to RANCH” by treating them as ligands bound to RNaseE. All truncated degradosome protomers in the four “monomer” pools were subsequently converted from invisible “hetero atom” C-α models back to visible “all atom” models with FULCHER, resulting in a pool of monomeric truncated degradosome protomers (Supplementary Figure S4b). The four monomer pools were then combined randomly with CombinerIM (Shkumatov et al, in preparation), which uses CRYSOL to calculate an in silico intensity profile for each member of the filtered truncated degradosome pool (Supplementary Figure S4d; Svergun et al., 1995). A pool of 1 million random truncated degradosome structures was then used as input for a genetic algorithm (GAJOE) that selects ensembles of structures/intensity profiles from the random pool in order to explain the experimental SAXS curve (Supplementary Figure S4e). *GAJOE* was run 10× to obtain an ensemble of models that best describes the experimental SAXS data.

### 4.9 In vitro transcription (IVT)

A 263-bp template encoding *E. coli* 9S RNA was amplified from pKK233-2 (provided by Dr. AJ Carpousis) using PCR (primers are depicted in Table 4). The PCR reaction was carried out with Phire Hot Start II polymerase (Finnzymes) according to the manufacturer’s protocol. Incorporated in one of the primers was a T7 RNA polymerase recognition sequence (underlined). PCR products were checked on a 1% agarose gel and purified with the QIAquick PCR Purification Kit (QIAGEN). The PRC product was used as a template for IVT.

IVT reactions were carried out in 200 μl volumes according to standard protocol. Each reaction was set up with 3.5 μg of template, and additionally supplemented with T7 RNA polymerase and DMSO (3% (v/v)). The IVT reaction was run at 37°C for 5 hr, and TURBO DNase I (Invitrogen) was added in the last 30 min to digest the DNA template. Transcribed 9S rRNA was then gel-purified from a 4% polyacrylamide gel. Bands containing RNA were visualized by UV–shadowing and excised. RNA was recovered from gel slices by overnight electroelution at 100 V in 1×TBE buffer using an EluTrap System (Whatman). Finally, RNA was purified with an RNA cleanup kit (Thermo Fisher Scientific) and the concentration was measured with a NanoDrop ND-1000 spectrophotometer (Thermo Fisher Scientific). Purified 9S rRNA was stored in MilliQ water at –20°C.

### 4.10 Ultracentrifugation

Based on the GraFix protocol (Kastner et al. 2008; Stark, 2010), the truncated degradosome was purified in Hepes buffer (50 mM HEPES pH 7.5, 400 mM NaCl, 100 mM KCl, 5 mM MgCl_2_, 1 mM TCEP, 0.02%β-DDM, 10% (v/v) glycerol) and diluted to 5 μM. 200 pmoles of truncated degradosome were loaded on a continuous glycerol/glutaral-dehyde gradient (10%–40% (v/v) glycerol) in an ultra-centrifugation tube and spun at 68,000 rcf for 18 hr, 4°C. The continuous gradient was made with a Gradient Master™ base unit (BioComp) according to the manufacturer’s protocol, and buffer S6-Hepes was used as a base buffer. The gradient was then carefully fractionated manually into 200 μl fractions (bottom to top) using a peristaltic pump at low flow rate as to not mix fractions in the pump tubing. The latter were used to detect fractions that contain truncated degradosome via standard dot blots or TCA precipitation.

### 4.11 Liposome extrusion and truncated degradosome reconstitution

To prepare liposomes that mimic the cytosolic membrane of *E. coli*, 1 mg of DOPE/DOPG was prepared by mixing 70% v/v of DOPE (Avanti Polar Lipids) with 30% v/v of DOPG (Avanti Polar Lipids). Lipids were dissolved in chloroform, mixed in proportion, dried overnight in a desiccator, and purged with Argon. 500 μl of liposome buffer (50 mM Tris-HCl pH 7.5, 50 mM NaCl, 150 mM KCl, 5 mM MgCl_2_, 1 mM TCEP) was added to resuspend lipids, which were left at 4°C for 40 min for hydration. Liposomes were prepared by extrusion with a Mini-Extruder (Avanti Polar Lipids, 20 passes) using membranes with 0.05 μm diameter pores (Avanti Polar Lipids). Liposomes were stored in the fridge at 4°C for a maximum of 4 days.

To reconstitute the truncated degradosome on the liposomes, 50 μg of truncated degradosome was added to 100 μg of fresh liposomes (200 μl total volume) and dialyzed overnight (4°C) against a detergent-free buffer supplemented with 100 mM glucose. Ultracentrifugation assays were carried out as described above. A 10%–40% glycerol gradient was set up and ultracentrifugation was carried out at 84000×g in a SW60 Ti rotor for 12 hr, 4°C. Fractions were precipitated with TCA and analyzed with SDS-PAGE.

Reconstitution of the full RNA degradosome was carried out as described for the truncated degradosome.

### 4.12 Electrophoretic mobility shift assays (EMSA)

EMSAs were used to assess interactions between the truncated degradosome/recognition core and the 30S ribosomal subunit. Binding reactions were performed at room temperature in binding buffer (40 mM Tris-HCl pH 7.5, 100 mM NaCl, 100 mM KCl, 10 mM MgCl_2_, 5 mM NH_4_Cl_2_, 1 mM TCEP). After a 15 min of incubation loading buffer (10% (w/v) sucrose) was added to the samples. Samples were loaded on a 0.6% agarose gel (agarose (Sigma-Aldrich) dissolved in 24 mM HEPES, 190 mM Glycine, 10 mM MgCl_2_ at pH 8.3) and run for 6 hr at 4°C in running buffer (24 mM HEPES, 190 mM Glycine, 10 mM MgCl_2_ at pH 8.3) with an applied electric field of 80 V. Sybrgold staining (Invitrogen) was used to stain the gel for RNA visualization, Coomassie staining was used for visualization of protein bands.

### 4.13 Cryo-EM, single particle analysis

The RNase E catalytic core (RNase E (1-529)) was concentrated to 10 μM and supplemented with 8 mM CHAPSO immediately before grid preparation. Grids were prepared following the procedure described above (R2/2 Quantifoil grids). A dataset was collected on a 300 kV Titan Krios (FEI, BioCem, Department of Biochemistry, University of Cambridge), and processed in Relion 2.1 (Nakane et al., 2018; Scheres, 2012; Zivanov et al., 2018) following standard protocol. After 3D refinements, the particles were transferred to CryoSparc 2.15.0 (Punjani et al., 2017) and subjected to another round of refinement. Prior to 3D variability analysis (3DVA) in CryoSparc, the particle set was expanded along its pseudo-C2 symmetry axis to allow for symmetry-breaking conformational heterogeneity to be modelled. 3DVA was run on a set of 64,000 symmetry expanded particles, solving for 6 modes of variability at a resolution cutoff of 8 Å. The three top variability modes were retained.

The recognition core was co-purified with the 30S small ribosomal subunit via size exclusion chromatography (Superose 6 10/300 GL column, GE Healthcare). Fractions were analyzed on an SDS-page gel and those containing the 30S-recognition core complex were concentrated using 15 ml Amicon Ultra 30,000 MWCO concentrator (Millipore). The concentration was measured using a NanoDrop ND-1000 spectrophotometer (Thermo Scientific) and a *λ*_260nm_ extinction coefficient of 13,394,967 M^–1^ cm^–1^. The complex (0.5 μM) was cross-linked with 0.2% of Glutaraldehyde (Sigma-Aldrich) for 30 min at room temperature. The cross-linking reaction was quenched with 10 μl of Tris-HCl pH 7.5. In addition, samples that were not cross-linked were prepared for cryoEM as well. Glow discharged gold grids (R1.2/1.3 Quantifoil) were prepared on a Vitrobot Mark IV (FEI) and screened with a 200 kV Talos Arctica microscope (BioCem, Department of Biochemistry, University of Cambridge, UK). A data collection was set up for a crosslinked sample on 200 kV Talos Arctica microscope (Falcon III, counting mode). Pre-processing (motion correction, ctf estimation and particle picking/extraction) were carried out on 1,400 movies in WARP 1.06 (Tegunov & Cramer, 2019). A particle set of 796 418 particles was then transferred to CryoSparc 2.15 (Punjani et al., 2017) for further processing. After several rounds of 2D classification, iterative heterogenous refinement was carried out to better resolve the density for the recognition core. 30,280 particles were used for a final round of 3D refinement, leading to a 9 Å reconstruction (GS-FSC). To better visualize the relatively weak density corresponding to the recognition core, the map was blurred with a b-factor of 700 Å^2^. A negative control dataset, i.e. the 30S small ribosomal subunit on its own, was collected as well and processed following the same procedure. 98,183 particles were picked from 121 micrographs, of which 23,378 were used for the final 3D reconstruction, at 5.5 Å (GS-FSC).

### 4.14 Fiducial marker preparation for cryoET

Gold fiducial markers (10 nm diameter, BBI Solutions, EMC10) were coated with bovine serum albumin (BSA) manually prior to cryoET grid preparation. 975 μl of BBI gold beads were buffered with 25 μl of 200 mM NaH_2_P0_4_ pH 5.0, after which 50 μl of a 5 mg/ml BSA stock (in 5 mM NaH_2_P0_4_ pH 5.0) was added. The mixture was incubated overnight at 4°C with gentle mixing. BSA-coated gold beads were collected by centrifugation (1 hr, 50,000 rcf, 4°C). The supernatant was removed, and gold beads were resuspended in liposome buffer (50 mM Tris-HCl pH 7.5, 50 mM NaCl, 150 mM KCl, 5 mM MgCl_2_, 1 mM TCEP). Gold beads were collected again by another round of centrifugation (1 hr, 50,000 rcf, 4°C) and resuspended in ~20 μl of liposome buffer. Gold beads were added to the cryoET samples in a 1:10 volumetric ratio (gold beads: sample).

### 4.15 Cryo-electron tomography and subtomogram averaging

Samples of proteoliposomes with truncated degradosome, prepared as described above, were dialyzed and a small aliquot analyzed by SDS-PAGE to check the integrity of the C-terminal domain of RNase E. 0.5–1 μl of BSA coated gold fiducials were mixed with 10 μl of proteoliposome sample, which was prepared as described above. The sample was then loaded onto glow discharged R2/2 Quantifoil grids with a Vitrobot Mark IV (FEI, BioCem, Department of Biochemistry, University of Cambridge, UK). Grids were screened with a 200 kV Talos Arctica transmission electron microscope (BioCem, Department of Biochemistry, University of Cambridge, UK). The 30S-proteoliposome samples and grids were prepared as described above, but the 30S small ribosomal subunit was added to the truncated degradosome-liposome sample in the last hour of the dialysis step, to allow for interactions with the membrane bound truncated degradosome.

Tilt series images were collected on a 200 kV Talos Arctica with Tomo4 (FEI) and on a 300 kV Titan Krios with Tomo5 (FEI) (BioCem, Department of Biochemistry, University of Cambridge, UK). All tilt series were collected from –60° to +60° with 3° increment, with image tracking before and after every tilt image, and autofocus for each image. On the Talos Arctica, images were collected with a Falcon III detector in linear mode (FEI) at a nominal pixel size of 2.29 Å/pix, and a bidirectional tilt scheme was followed, with a total dose of 99 e/Å^2^ per tilt series. On the Titan Krios movies were collected (15 frames) with a K3 camera in counting mode (Gatan) at a nominal pixel size of 1.43 Å/pix. A single step dose-symmetric tilt scheme was used, with a total dose of 125 e/Å^2^ per tilt series. For the full RNA degradosome bound to liposomes images at 0° tilt were collected only.

The tilt series of the membrane-bound truncated degradosome were aligned in Etomo (part of the IMOD package, Kremer et al., 1996). After coarse alignment of the tilt series, a fiducial model was picked and refined manually. Using the refined fiducial model, tilt series were aligned both globally and locally. Eight tomograms (binned by a factor of 2) were reconstructed from eight aligned tilt series via weighted back projection (WBP), using a SIRT-like (simultaneous iterative reconstruction technique) filter for visualization purposes. To plot the averaged radial density profile of the truncated degradosome on the membrane, spherical liposomes coated with truncated degradosome were centered and cropped in Dynamo from CTF corrected, unfiltered tomograms binned to 8.8 Å/pix (Castaño-Díez et al., 2012, 2017). A MATLAB script was written to calculate and plot the radial density profile of these sub-tomograms, i.e. the average density profile from the center of the volume to the edge. The script applies an icosahedral symmetry operator to the volume, followed by fine rotational averaging over the X- and Y-axes in Dynamo.

Sub-tomogram averaging of the 30S small ribosomal subunit was carried out following two different strategies. Tilt series collected on the Talos Arctica were aligned in IMOD as described above. Global/local alignments and gold fiducial positions were transferred to emClarity (Himes & Zhang, 2018) for 2D- and 3D-CTF correction, template matching and sub-tomogram extraction, and sub-tomogram alignment/averaging. For template matching, a reference map was generated in UCSF Chimera from the 30S structure using pdb 4ADV (Pettersen et al., 2004) and scaled with emClarity. Template matching and the first rounds of alignment/averaging were carried out on 4x binned tomograms (9.16 Å/pix). The angular searches and shifts of the alignments were refined gradually, and binning was reduced (4.58 Å/pix). Two rounds of TomoCPR were included to refine the tilt-series alignments based on aligned 30S particles. A sub-tomogram average of the 30S small ribosomal subunit was reconstructed at 11.7 Å, which is close to Nyquist (9.16 Å), after 17 rounds of alignments. The 30S average was then mapped back into the whole tomogram, preserving its original orientation and replacing the noisy sub-tomogram volumes of the 30S small ribosomal subunit particles. In parallel, TomoSegmemTV was used to model the lipid membranes in the raw tomogram. Together, these two operations present the spatial distribution of the 30S small ribosomal subunits in the tomogram and their position/pose with respect to the lipid membranes, which was visualized in UCSF Chimera.

A different approach was used for the tilt series of 30S-proteoliposomes collected on a Titan Krios (Thermo Fisher Scientific). Movies were preprocessed in WARP (Tegunov & Cramer, 2019) (Motion correction and ctf estimation). mdocspoofer (https://github.com/alisterburt) was used to generate mdoc metadata files per tilt series. These mdoc files are used by Warp to generate preprocessed and ordered stacks per tilt series. These tilt series were then imported in Dynamo (Castaño-Díez et al., 2012) and aligned with dautoalign4warp (Burt et al., 2021), a MATLAB script that performs automated tilt series alignment in Dynamo. The tilt series alignments were imported into Warp for robust 3D-CTF estimation and tomogram reconstruction. Each tomogram was reconstructed twice, once unfiltered and once deconvolved (for visualization). The same 30S template as described above (scaled to 1.43 Å/pix) was used for template matching in WARP. The template and tomograms were binned to 10.01 Å/pix on the fly during template matching, and picked coordinates were reconstructed as sub-tomogram volumes at 5.72 Å in Warp, together with a 3D-CTF volume. Metadata and initial poses were recorded in a Relion.star file. The extracted sub-tomogram volumes were then imported into Relion 3.0.8 for 3D classification and 3D refinement. A clean set of particles were then imported in *M* (Tegunov et al., 2021) for further refinement of the 30S sub-tomogram averages and local refinement of the tilt-series alignments, Ctf refinements, and magnification correction. Sub-tomograms were unbinned and refined once more with *M*, resulting in a reconstruction at 7.5 Å resolution (GS-FSC).

## Supplementary Material

Supplementary Material

## Figures and Tables

**Table 1:** A non-uniform charge distribution across RNA degradosome components

Protein or segment	Identification code uniprot	Isoelectric point, protomer
RNase E	P21513	5.47
RNase E catalytic domain 1-529, and with membrane interaction site 1-602	P21513	6.16, 6.01
RNase E RNA binding region 603-850; PNPase interaction segment 851-1063	P21513	10.67, 4.11
RhlB, RecA core 1-390, and C-terminal tail, 391-421	POA8J8	7.29, 5.98, 12.54
PNPase	PO5O55	5.10
Enolase	POA6P9	5.32

*Note:* The isoelectric points for the degradosome components were estimated using ProtParam. While most segments are electronegative, there are strongly basic portions in the C-terminal tail of RhlB (highlighted in green) and the RNA binding segment and recruitment site for enolase and RhlB of RNase E, 603-850 (highlighted in red). The electropositive region is expected to be clustered spatially, and in the absence of bound RNA, to have favorable interactions with the negatively charged head groups of the membrane.

**Table 2:** RNase E (1-850) mutagenesis primers

Primer name	Primer sequence (5′-3′)
RNaseEEcoFor – 1	CCAGGATCCGAATTCGATGAAAAGAATGTTAATCAACG
RNaseE850RevSal – 4	GCACAGGAACAATGGCGTGAACTTCCTGGGTG
NTD_F – 3	CACCCAGGAAGTTCACGCCATTGTTCCTGTGC
NTD_R – 2	CGCAAGCTTGTCGACTTACTCAACAGGTTGC

**Table 3:** RNase E (1-1064) mutagenesis primers

Primer name	Primer sequence (5′-3′)
RnePrsfF – 1	CCAGGATCCGAATTCGATGAAAAGAATGTTAATCAACG
RneIntR – 4	GCACAGGAACAATGGCGTGAACTTCCTGGGTG
RneIntF – 3	CACCCAGGAAGTTCACGCCATTGTTCCTGTGC
RnePrsfR – 2	CGCAAGCTTGTCGACTTACTCAACAGGTTGC
pRSF1-InFusion	GAATTCGGATCCTGGCTGTGGTGATG
pRSF2-InFusion	GTCGACAAGCTTGCGGCCGCATAATGC

**Table 4:** 9S template amplification primers for in vitro transcription

Primer	Primer sequence (5′-3′)
9SForNew	GTTTTTAATACGACTCACTATAGAAGCTGTTTTGGCGGATGAGAGAAG
9SRev	CGAAAGGCCCAGTCTTTCGACTGAGC

## Data Availability

The data that support the findings of this study are available from the corresponding author upon reasonable request.
