# Supplementary material for: Multi-scale ensemble properties of the Escherichia coli RNA degradosome
Source: Mol Microbiol. Author manuscript; Available in PMC 2022 Aug 9. (PMC7613265; doi:10.1111/mmi.14800)
Supplement: Supplementary Material [file EMS152041-supplement-Supplementary_Material.pdf]

Supplementary Material

Multi-scale ensemble properties of the *Escherichia coli* RNA degradosome

Tom Dendooven, Giulia Paris, Alexander V. Shkumatov *, M.S. Islam, Alister Burt**, Marta A. Kubańska***, Tai Yuchen Yang, S.W. Hardwick, Ben F. Luisi

Department of Biochemistry, University of Cambridge, Tennis Court Road, Cambridge CB2 1GA, U.K.

*Center for Structural Biology, Vlaams Instituut voor Biotechnologie, Pleinlaan 2, Brussels 1050, Belgium, and Structural Biology Brussels, Department of Bioengineering Sciences, Vrije Universiteit Brussel, Pleinlaan 2, Brussels 1050, Belgium.

**Institut de Biologie Structurale, Université Grenoble Alpes, CEA, CNRS, IBS, 71 Avenue des martyrs, F-38044 Grenoble, France

Correspondence : bfl20@cam.ac.uk

***Current address: Department of Molecular Sociology, Max Planck Institute of Biophysics, Max-von-Laue-Str. 3, 60438 Frankfurt am Main, Germany

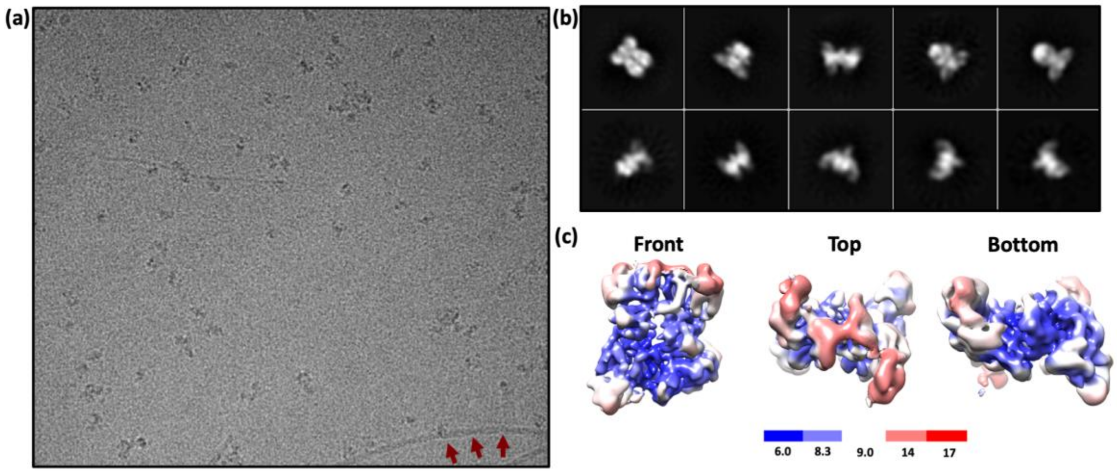

Supplementary Figure S1. **Overview of the RNase E (1-529) cryo-EM experiments**. **(a)** A representative cryo-EM image at 3 μm defocus. Red arrows point to a CHAPSO fibre. **(b)** 2D class averages reveal homogeneous particles with apparent cyclic symmetry (C2). **(c)** Local resolution estimation of the cryo-EM map presented in Figure 2A. The top side of the cryo-EM reconstruction is resolved significantly worse than the bottom side.

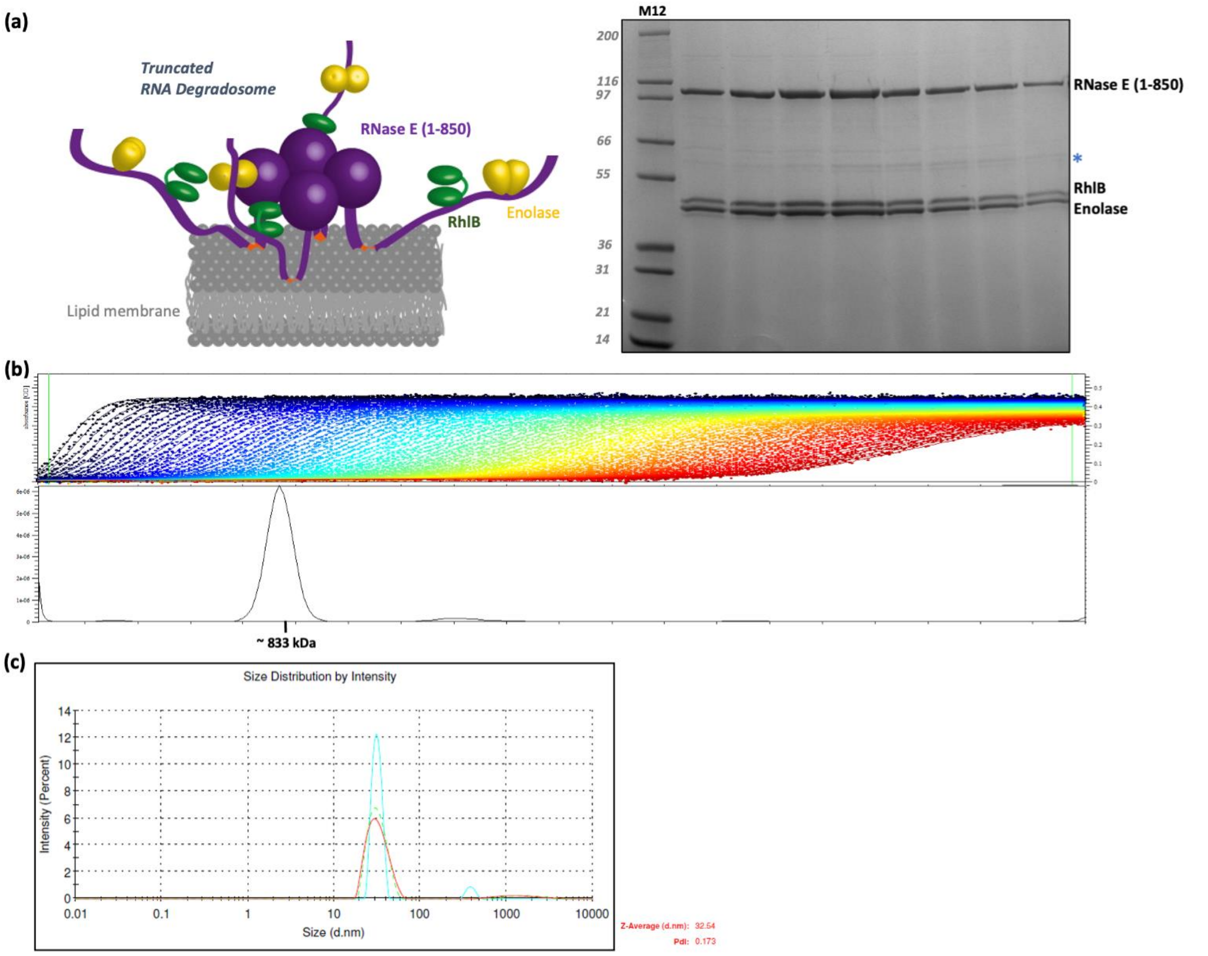

Supplementary Figure S2. **Purification and biophysical analysis. (a)** Schematic and SDS-PAGE analysis of the purified in vivo reconstituted truncated degradosome. The * corresponds to degradation product. Schematic adapted from Bandyra *et al.* (2012)**. (b)** Analytical ultracentrifugation sedimentation velocity experiments reveal a homogenous truncated degradosome with a molar mass estimated at ~833 kDa. A frictional ratio of 1.48 was calculated, indicating that the truncated degradosome is elongated. Absorbance profiles during ultracentrifugation are shown in the top panel and molar mass distribution is shown in the bottom panel. **(c)** Dynamic light scattering experiments indicate that the truncated degradosome is homogenous and has a hydrodynamic size of 325 Å. DLS experiments were performed in triplicate.

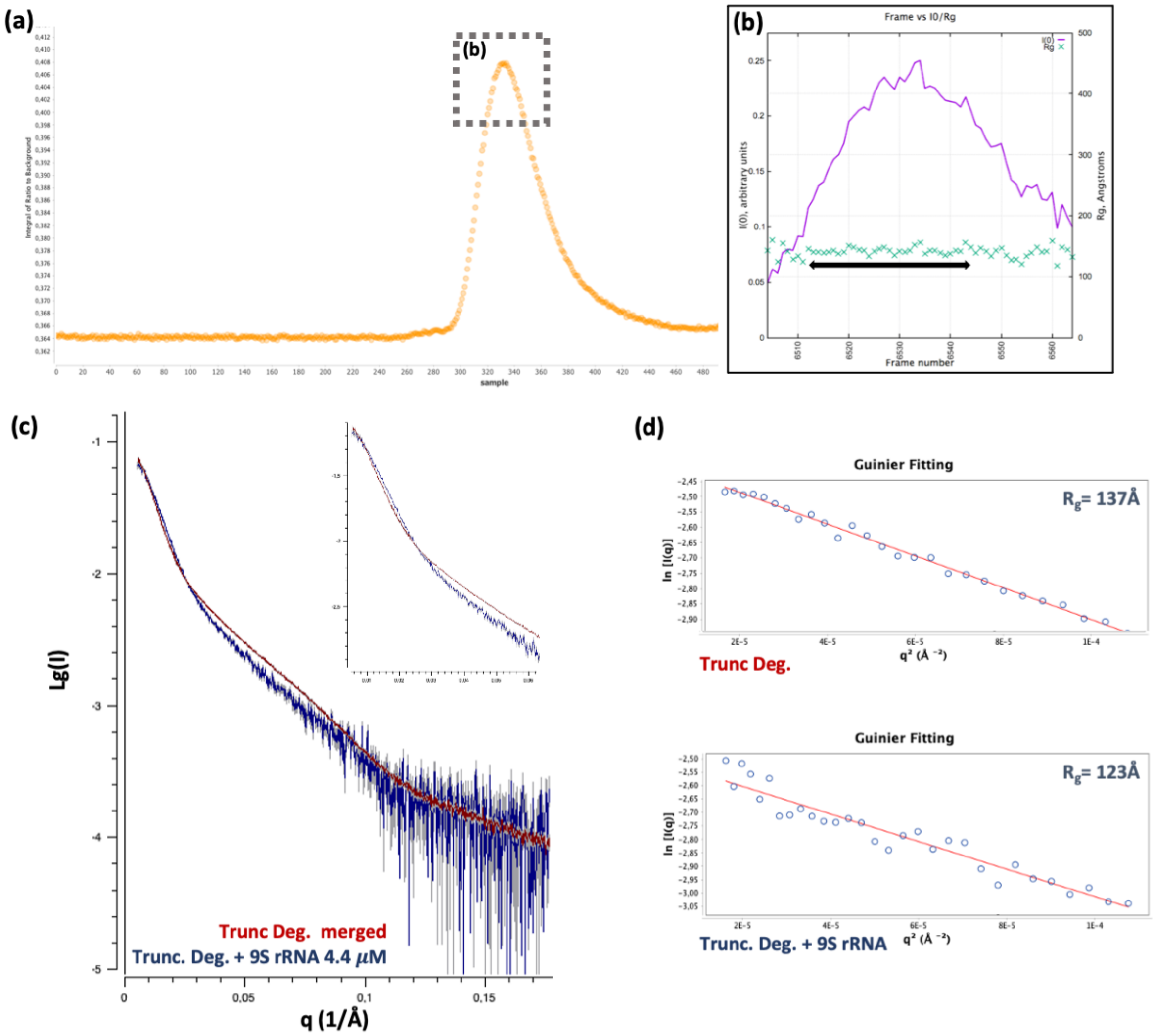

Supplementary Figure S3. **SEC-SAXS analysis of the truncated degradosome.** **(a)** The SEC profile from SEC-SAXS experiments shows that the truncated degradosome elutes as a single peak. The SEC profile depicted here and in (b) corresponds to a 14.4 μM truncated degradosome sample. **(b)** The radius of gyration, R_g, as monitored over the elution peak with dataSW (Shkumatov & Strelkov, 2015), shows that the truncated degradosome assembly has a high degree of compositional homogeneity. The double arrow marks the frames that were used to generate the final intensity curve. **(c)** Radially averaged and buffer subtracted intensity profiles for the truncated degradosome sample (red curve, merged from four different concentrations (2.2 μM, 4.4 μM, 7.2 μM, 14.4 μM) and the truncated degradosome bound to 9S rRNA (blue curve). Error bars are depicted in grey. **(d)** Guinier plots and calculated Rg (123 Å) for the curves in C.

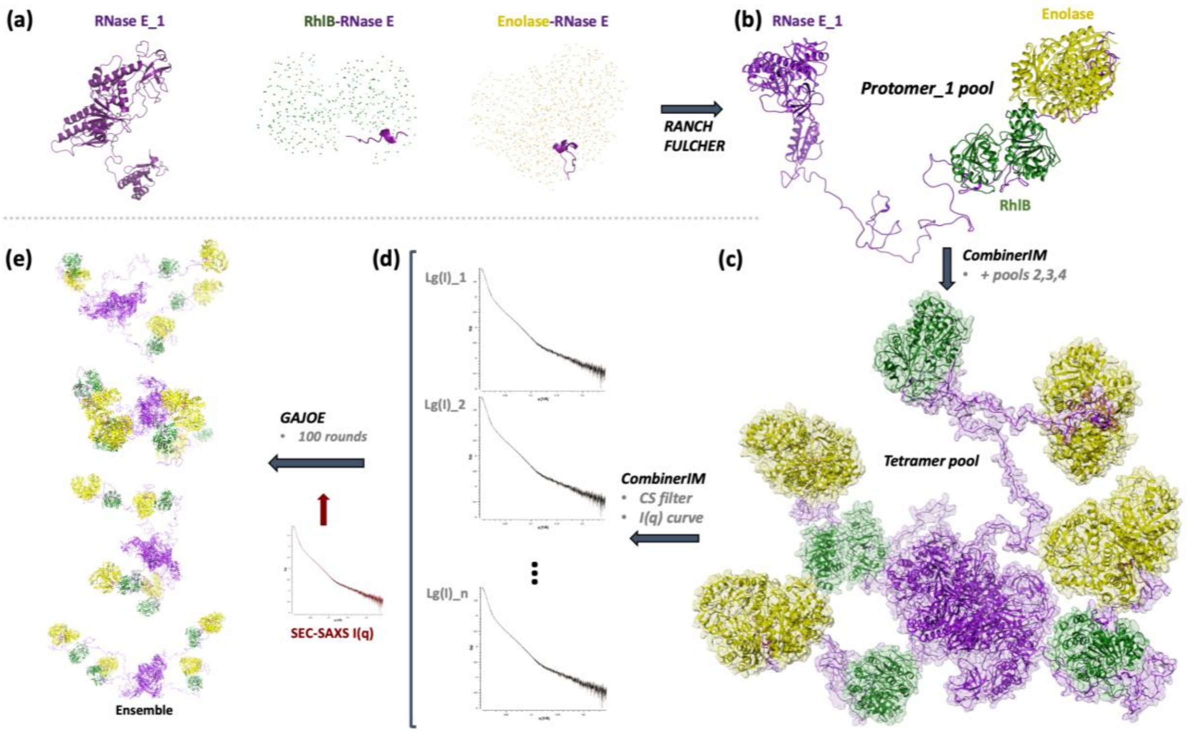

Supplementary Figure S4. **Schematic overview of the tailored EOM pipeline used for the truncated degradosome. (a)** Prior knowledge about truncated degradosome components and their interactions with the RNase E scaffold domain was combined in the form of a crystal structure (RNase E, purple), a co-crystal structure (Enolase, yellow) and a homology model (RhlB, Green). RhlB and Enolase were made ‘invisible’ to RANCH and are presented as dots. **(b)** RANCH was used to generate random truncated degradosome protomers based on (a), modelling the RNase E scaffold domain as an intrinsically disordered peptide. FULCHER was used to convert Enolase and RhlB back to all-atom models. For each truncated degradosome protomer a random ‘monomer’ pool of 20,000 structures was generated. **(c)** CombinerIM was used to combine protomers of each pool to generate random structures of tetrameric truncated degradosome assemblies. A pool of 10,575,160 random truncated degradosome structures was generated. **(d)** Next, CombinerIM was used to filter the random truncated degradosome pool based on a clash score threshold (CS filter) and calculate in silico intensity profiles for each assembly with CRYSOL (Svergun et al., 1995). **(e)** This final pool of 2,920,079 intensity profiles was scanned with GAJOE to find ensembles that, when averaged together, fit the experimental intensity profile for the truncated degradosome.

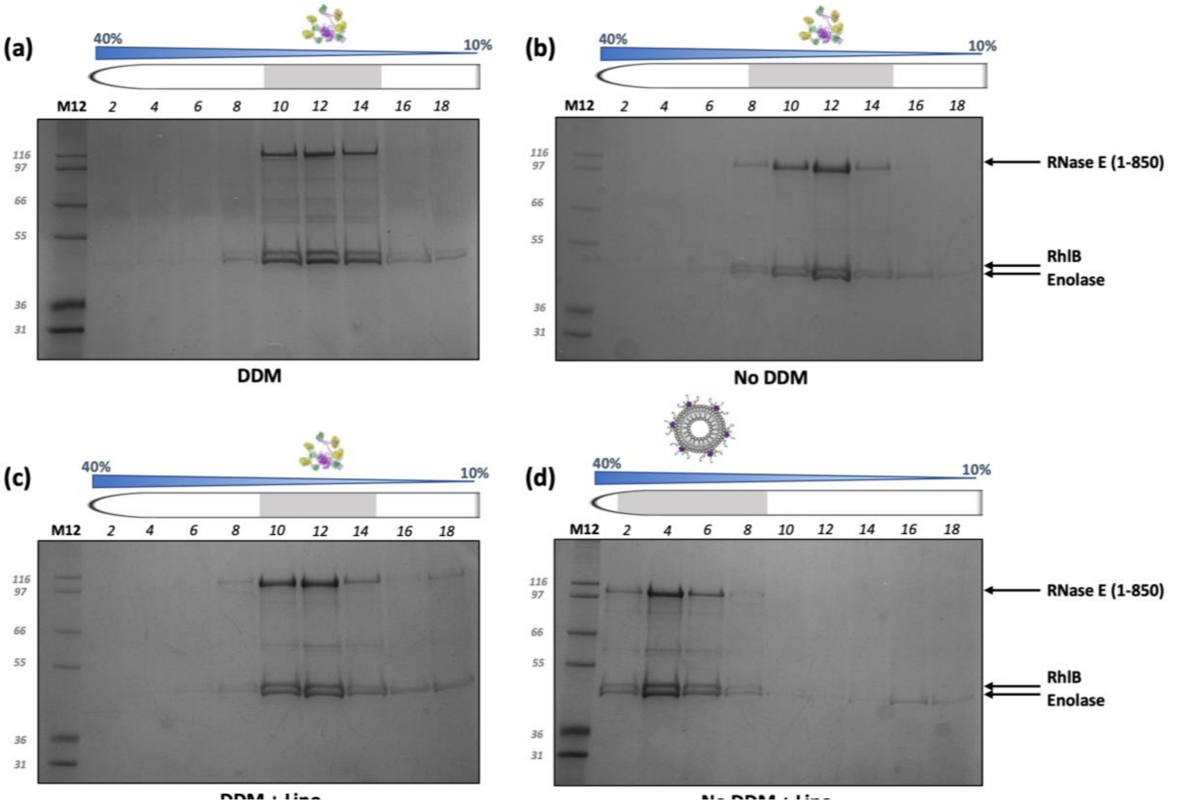

Supplementary Figure S5. Reconstitution of truncated degradosome on liposomes. **(a-b)** The truncated degradosome migrates close to halfway down the glycerol gradient in either the presence (a) or absence (b) of β-DDM. **(c)** When both beta-DDM and liposomes are present, the truncated degradosome does not adhere to the lipid membrane and migrates almost halfway down the glycerol gradient. **(d)** When β-DDM is dialysed out and liposomes are added to the sample, the truncated degradosome binds to the lipid membranes and migrates closer to the bottom of the tube, together with the lipid vesicles. Fractions are annotated on top of each gel. Molecular weights of the protein standards are annotated on the left of each gel. Lipo, liposomes; DDM, β-DDM (0.02% w/v); M12, Mark 12 protein ladder.

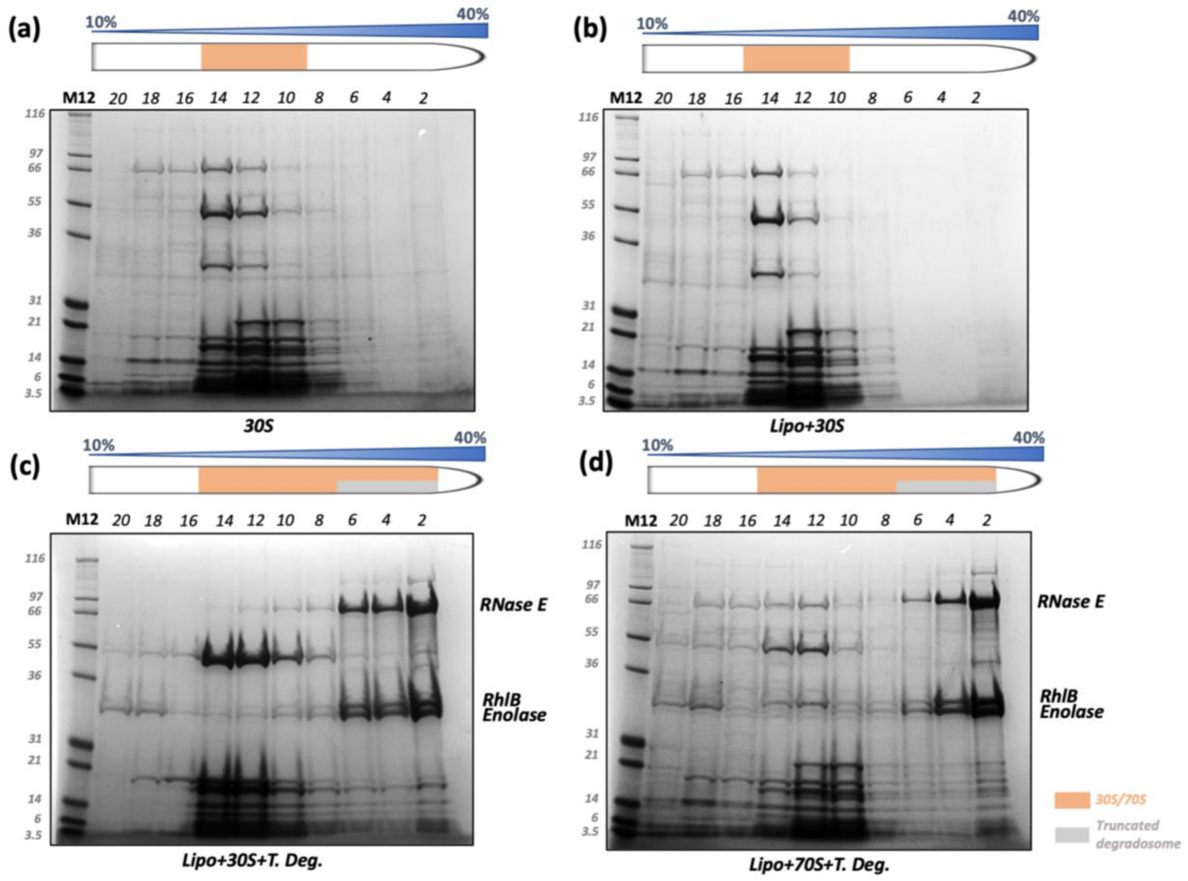

Supplementary Figure S6. Ultracentrifugation assays of the membrane-associated degradosome with the 70S ribosome and 30S small ribosomal subunit. **(a)** The 30S small ribosomal subunit migrates halfway down the glycerol gradient during the ultracentrifugation step. **(b)** The 30S small ribosomal subunit does not adhere to lipid membranes and migrates halfway down the glycerol gradient when liposomes are present in the sample. **(c)** When the truncated degradosome is bound to the lipid vesicles, a fraction of the 30S small ribosomal subunit co-migrates down the glycerol gradient with the liposomes. **(d)** Same as (c) but for the 70S ribosome. Fractions and glycerol gradients (v/v %) are annotated on top of each gel. Molecular weights of the protein standards are annotated on the left of each gel. Lipo, liposomes; T. Deg., truncated degradosome; M12, Mark 12 protein ladder.

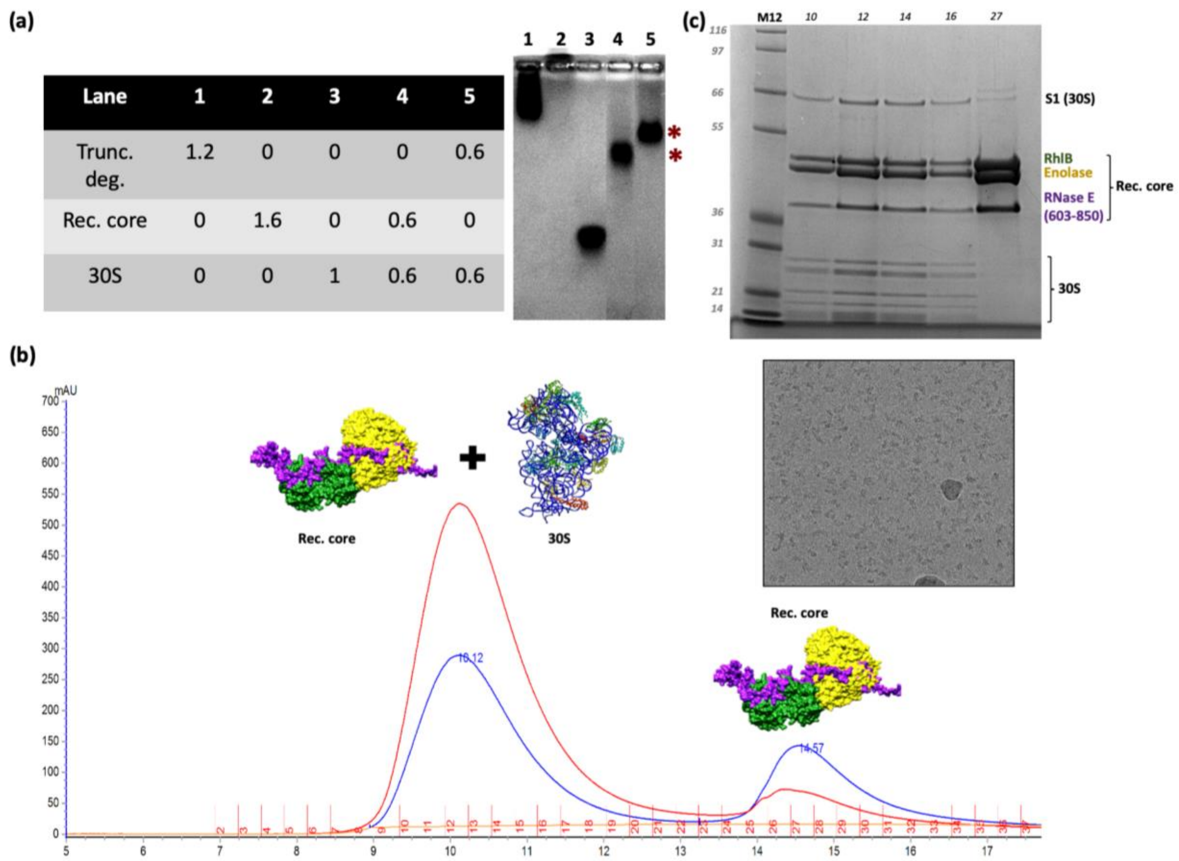

Lane	1	2	3	4	5
Trunc. deg.	1.2	0	0	0	0.6
Rec. core	0	1.6	0	0.6	0
30S	0	0	1	0.6	0.6

Supplementary Figure S7. Electrophoretic mobility shift assays (EMSA) and co-purification of putative surveillance super-complexes. **(a)** EMSA indicating that 30S-recognition core and 30S-truncated degradosome super-complexes can form in solution (*). Values in the table are in μM. **(b)** Co-purification of the 30S-recognition core super-complex via size exclusion chromatography results in two main peaks, one of which has a strong 260 nm absorbance, as expected for the 30S component in the complex. RNase E (603-850) is in purple, RhlB in green and Enolase in yellow. The inset shows a representative cryo-EM image of the recognition core-30S super complex. **(c)** SDS-PAGE analysis of peak fractions reveals that the recognition core co-purifies with the 30S ribosome (lanes 10-16). Fraction 27, corresponding to the second, smaller peak in the elution profile contains excess recognition core. 30S, small ribosomal subunit; Rec. core, Recognition core.
